# The Isotropic Material Design of In-Plane Loaded Elasto-Plastic Plates

**DOI:** 10.3390/ma14237430

**Published:** 2021-12-03

**Authors:** Sławomir Czarnecki, Tomasz Lewiński

**Affiliations:** Faculty of Civil Engineering, Warsaw University of Technology, 00-637 Warszawa, Poland; t.lewinski@il.pw.edu.pl

**Keywords:** isotropic material design, compliance minimization, Hencky-Nadai-Ilyushin plasticity

## Abstract

This paper puts forward a new version of the Isotropic Material Design method for the optimum design of structures made of an elasto-plastic material within the Hencky-Nadai-Ilyushin theory. This method provides the optimal layouts of the moduli of isotropy to make the overall compliance minimal. Thus, the bulk and shear moduli are the only design variables, both assumed as non-negative fields. The trace of the Hooke tensor represents the unit cost of the design. The yield condition is assumed to be independent of the design variables, to make the design process as simple as possible. By eliminating the design variables, the optimum design problem is reduced to the pair of the two mutually dual Linear Constrained Problems (LCP). The solution to the LCP stress-based problem directly determines the layout of the optimal moduli. A numerical method has been developed to construct approximate solutions, which paves the way for constructing the final layouts of the elastic moduli. Selected illustrative solutions are reported, corresponding to various data concerning the yield limit and the cost of the design. The yield condition introduced in this paper results in bounding the values of the optimal moduli in the places of possible stress concentration, such as reentrant corners.

## 1. Introduction

The problem of designing structures made of a linear elastic material is one of the major topics of Free Material Design (FMD). Within this approach, all the elastic moduli of Hooke’s tensor C are design variables. Usually, the aim is to minimize the compliance of the structure, while the unit cost is identified with the trace of Hooke’s tensor or the sum of its eigenvalues (see [1,2], to mention the first papers on the topic). The additional assumption of isotropy reduces the number of design variables to two: the bulk modulus *k* and shear modulus *µ* (see [3,4], where this method, called there the Isotropic Material Design (IMD), was proposed). In the 3D setting, the eigenvalues of the Hooke tensor are: 3k,2μ,2μ,2μ,2μ,2μ, hence tr C=3k+10μ. In the 2D setting, the eigenvalues of **C** are: 2k,2μ,2μ, hence tr C=2k+4μ. The present paper refers to those papers on FMD in which the Hooke tensor is subject only to the condition of positive semi-definiteness; in the case of the IMD method, this condition reduces to: k≥0,μ≥0. The upper bounds are absent to make the theory as simple as possible. Admitting the vanishing of moduli means working with the broadest possible class of the underlying microstructures. For instance, the hexagonal (or honeycomb in the plane) gridwork is characterized by a very small shear modulus, if ligaments are slender (see [5,6]). On the other hand, spiral microstructures are characterized by very small bulk modulus, which implies the effective Poisson ratio almost attaining its lower 2D limit equal −1 (see [7,8,9,10,11,12]). To encompass such a broad class of composites, it is necessary to admit the largest possible range of the bulk and shear moduli. Due to the simplicity of such modeling, it is possible to perform minimization over the moduli analytically, thus eliminating the design variables in the first step. Eventually, one arrives at two, mutually dual, linear constrained problems (LCP) in the meaning of Bouchitté and Fragalà [13]. The primal LCP problem is stress-based, with the integrand expressed by a norm: ρ(⋅); its minimizer determines the effective moduli directly. The dual one is displacement-based and leads to the locking of strains within the unit ball defined by $\rho^{o}\left(\cdot \right)$, the function polar to ρ(⋅). The stress field, which solves the stress-based LCP problem, is simultaneously the stress field emerging in the optimal structure. On the other hand, the displacement field of the second LCP problem is proportional to the displacement field of the optimal structure. In the IMD optimal structure, the values of stresses are proportional to the values of the bulk and shear moduli, since the values of strains are subjected to the locking conditions $\rho^{o}\left(\varepsilon \right)\leq 1$. The mathematical proofs of these interrelations can be found in [14].

If the support conditions have discontinuities or the domain has reentrant corners, then the stress fields assume infinite values around these points. Consequently, the values of the optimal bulk or shear moduli may tend to infinity. The aim of the present paper is to cut these extremes of the plots of stresses. To this end, we impose the local yield conditions on the stress fields: $\gamma \left(\tau \right)\leq \sigma_{0}$, where γ(⋅) is a certain 1-homogeneous function and $\sigma_{0}$ represents a plastic limit corresponding to the tensile test. Such an approach is compatible with the Hencky-Nadai-Ilyushin theory of elasto-plastic structures. The stress-based problem of this theory differs from the stress-based problem of linear elasticity in the presence of the local yield condition $\gamma \left(\tau \right)\leq \sigma_{0}$ (see Chapter 5 in [15]).

The introduction of the plasticity limit essentially changes the IMD method, since bounding the stresses entails bounding the values of the optimal bulk and shear moduli. The novelty of the present paper is this concept of the IMD method in its elasto-plastic setting. 

In the present paper, the yield condition will not involve the design variables; both the function γ(⋅) as well as the bound $\sigma_{0}$ will be viewed as fixed during the optimization process. On one hand, the elastic moduli represent only elastic properties and they can be viewed as having nothing to do with the plasticity limit, like in the case of a continuum description of brick masonry structures (see comments in Section 3 in [16]) or in composites with relatively stiff grains embedded within a soft matrix. It is clear that mainly the plastic properties of the matrix determine the overall plastic response of the composite. On the other hand, in general, all the effective characteristics of composites reflect the internal properties of the underlying microstructure (i.e., all features of the representative volume element) (see [17]), which inevitably makes the design variables linked with the form of the plasticity limit. In the present paper, this link will not be taken into account.

The natural formulation of the statics problem within the theory by Hencky-Nadai-Ilyushin involves stresses as unknowns (see Chapter 5, Section 6.2 in [15]). The stress-based FEM was already developed in the 1970s (see [18]) and then extended to elasto-plasticity [19]. In the present paper, the present authors’ stress-based FE algorithm is proposed, originated in [20] in the context of Anisotropic Material Design, extended to the elastic IMD setting in [3,10,11] and adopted here to the elasto-plastic version of the IMD method. Since the numerical method of solving IMD problems plays an essential role here, it is put forward in detail in Section 5, while the numerical optimization procedure is explained in Section 6. 

This paper does not concern the problem of the reconstruction of the microstructure whose effective properties would correspond to the optimum design proposed. Such reconstruction can be performed using the tools developed by Suquet in [17], which will be the subject of forthcoming works.

The following notation and conventions are adopted. The *d*-dimensional design domain Ω is parameterized by the Cartesian coordinate system (*x*,*y*,*z*) for *d* = 3 and (*x*,*y*) for *d* = 2. The components of the displacement vector **u** will be denoted by $\left(u_{x},u_{y},u_{z}\right)$ for *d* = 3 and $\left(u_{x},u_{y}\right)$ for *d* = 2. In the 3D case (*d* = 3), the components of 2nd rank tensors of stress and strain in the (*x*,*y*,*z*) framework form the matrices:

(1) $$\sigma \sim \left[\begin{array}{ccc} \sigma_{x} & \sigma_{xy} & \sigma_{xz}\\ \sigma_{yx} & \sigma_{y} & \sigma_{yz}\\ \sigma_{zx} & \sigma_{zy} & \sigma_{z} \end{array}\right],\;\varepsilon \sim \left[\begin{array}{ccc} \varepsilon_{x} & \varepsilon_{xy} & \varepsilon_{xz}\\ \varepsilon_{yx} & \varepsilon_{y} & \varepsilon_{yz}\\ \varepsilon_{zx} & \varepsilon_{zy} & \varepsilon_{z} \end{array}\right]$$

The sign ~ means that the tensor is represented by the given matrix in the fixed Cartesian coordinate system. Both stress and strain tensors are symmetric. Let **I** represent the identity matrix, or **I** = diag [1,1,1]. The scalar product of two vectors **u**, **v** is defined by $u\cdot v=u_{x}v_{x}+u_{y}v_{y}+u_{z}v_{z}$. The set of 2nd rank symmetric tensors will be denoted by $E_{s}^{2}$. The scalar product of $\sigma ,\varepsilon \in E_{s}^{2}$ is defined by:

(2) $$\sigma \cdot \varepsilon =\sigma_{x}\varepsilon_{x}+2\sigma_{xy}\varepsilon_{xy}+\sigma_{y}\varepsilon_{y}+2\sigma_{xz}\varepsilon_{xz}+2\sigma_{yz}\varepsilon_{yz}+\sigma_{z}\varepsilon_{z}.$$

The Euclidean norms of the vectors and tensors in $E_{s}^{2}$ are denoted by $\left\|u\right\|=\sqrt{u\cdot u}$, $\left\|\sigma \right\|=\sqrt{\sigma \cdot \sigma }$. The trace of the tensor $\sigma \in E_{s}^{2}$ is given by $\mathrm{tr}\;\sigma =\sigma_{x}+\sigma_{y}+\sigma_{z}$. The deviator of $\sigma \in E_{s}^{2}$ is defined by:

(3) $$\mathrm{dev}\;\sigma =\sigma -\frac{1}{3}\left(\mathrm{tr}\;\sigma \right)\;I.$$

The Euclidean norm of the deviator reads:

(4) $$\left\|\mathrm{dev}\;\sigma \right\|=\sqrt{\frac{1}{3}\left[{\left(\sigma_{x}-\sigma_{y}\right)}^{2}+{\left(\sigma_{x}-\sigma_{z}\right)}^{2}+{\left(\sigma_{y}-\sigma_{z}\right)}^{2}+6\left(\sigma_{xy}^{2}+\sigma_{yz}^{2}+\sigma_{xz}^{2}\right)\right]}.$$

In the 2D case (*d* = 2), the tensors of stress and strain are represented by the matrices:

(5) $$\sigma \sim \left[\begin{array}{cc} \sigma_{x} & \sigma_{xy}\\ \sigma_{yx} & \sigma_{y} \end{array}\right],\;\varepsilon \sim \left[\begin{array}{cc} \varepsilon_{x} & \varepsilon_{xy}\\ \varepsilon_{yx} & \varepsilon_{y} \end{array}\right].$$

The identity matrix is defined by **I** = diag [1,1]. The trace of the tensor $\sigma \in E_{s}^{2}$ is given by $\mathrm{tr}\;\sigma =\sigma_{x}+\sigma_{y}$. The deviator of $\sigma \in E_{s}^{2}$ is defined by:

(6) $$\mathrm{dev}\;\sigma =\sigma -\frac{1}{2}\left(\mathrm{tr}\;\sigma \right)\;I$$

or

(7) $$\mathrm{dev}\;\sigma \sim \left[\begin{array}{cc} \frac{1}{2}\left(\sigma_{x}-\sigma_{y}\right) & \sigma_{xy}\\ \sigma_{yx} & \frac{1}{2}\left(\sigma_{y}-\sigma_{x}\right) \end{array}\right].\;$$

The Euclidean norm of the deviator reads:

(8) $$\left\|\mathrm{dev}\;\sigma \right\|=\sqrt{\frac{1}{2}{\left(\sigma_{x}-\sigma_{y}\right)}^{2}+2\sigma_{xy}^{2}}.\;$$

For both cases of *d* = 2 or *d* = 3, the scalar product of the two tensors from $E_{s}^{2}$ can be rewritten as:

(9) σ⋅ε=Tr σ ⋅Tr ε+dev σ⋅dev ε

where:

(10) $$\mathrm{Tr}\;\sigma =\frac{1}{\sqrt{d}}\mathrm{tr}\;\sigma$$

which is a modified trace of a tensor.

According to the linear theory of the continuum media, the strain tensor is the symmetric part of the gradient of the displacement vector. In the case of *d* = 2, we define the operation:

(11) $$\varepsilon \;\left(v\right)=\left[\begin{array}{cc} \frac{\partial v_{x}}{\partial x} & \frac{1}{2}\left(\frac{\partial v_{x}}{\partial y}+\frac{\partial v_{y}}{\partial x}\right)\\ \frac{1}{2}\left(\frac{\partial v_{x}}{\partial y}+\frac{\partial v_{y}}{\partial x}\right) & \frac{\partial v_{y}}{\partial y} \end{array}\right]$$

which determines the virtual strains corresponding to the virtual displacement field **v**. For a given function f(⋅) of argument $\sigma \in E_{s}^{2}$, one can define its polar by:

(12) $$\;\eta \;\in E_{s}^{2}\to f^{o}\left(\eta \right)=\max\left\{\eta \cdot \sigma |\;f\left(\sigma \right)\leq 1\;\right\}.$$

## 2. On the Hencky-Nadai-Ilyushin Theory of an Elasto-Plastic Body

Within the theory by Hencky-Nadai-Ilyushin (also called Hencky’s theory, see [15]), the stress state **σ** is locally constrained by the plasticity condition:

(13)  F(σ(x))≤0,  x∈Ω,

where Ω is the domain occupied by the body. The function *F* is assumed to be convex and continuous with respect to all stress components. Let us recall the HMH plasticity condition for isotropic metals proposed by Huber, Mises and Hencky (see [21]):

(14) $$\;\sigma_{eff}^{3D}\;\leq \sigma_{0}^{3D},\sigma_{eff}^{3D}=\sqrt{\frac{3}{2}}\left\|\mathrm{dev}\;\sigma \right\|,\;$$

where ‖dev σ‖ is given by Equation (4) and refers to the 3D setting, and $\sigma_{0}^{3D}$ is the plastic limit corresponding to the tensile test. Thus, the hydrostatic state of stress σ=pI cannot cause plastic yielding, irrespective of the sign of the pressure *p*. 

The present paper deals with the optimum design of in-plane loaded, transversely homogeneous thin plates of thickness *b*; Ω will be its middle plane parameterized by the (*x*,*y*) system. In such a plate, the stress components $\sigma_{z},\sigma_{xz},\sigma_{yz}$ are negligible in comparison to other stresses. Substituting: $\sigma_{z}=0,\sigma_{xz}=0,\sigma_{yz}=0$ into Equation (14) leads to the HMH condition for the plane-stress problem:

(15) $$\;\sigma_{eff}^{}\;\leq \sigma_{0},\sigma_{eff}^{}=\sqrt{\sigma_{x}^{2}-\sigma_{x}^{}\sigma_{y}^{}+\sigma_{y}^{2}+3\sigma_{xy}^{2}}.\;$$

Here, the stress resultants are involved, still denoted by $\sigma_{x},\sigma_{xy},\sigma_{y}$ of units N/m and $\sigma_{0}=b\sigma_{0}^{3D}$. It is worth noting that substitution $\sigma_{z}=0,\sigma_{xz}=0,\sigma_{yz}=0$ into Equation (4) does not lead to Equation (8). Indeed, Equation (15) now involves both stress invariants within the 2D setting, since:

(16) $$\;\sigma_{eff}^{}=\gamma \left(\sigma \right),\gamma \left(\sigma \right)=\sqrt{\frac{1}{2}{\left(\mathrm{Tr}\;\sigma \right)}^{2}+\frac{3}{2}{\left\|\mathrm{dev}\;\sigma \right\|}^{2}}$$

with $\mathrm{Tr}\;\sigma =(\mathrm{tr}\;\sigma )/\sqrt{2}\;\mathrm{and}\;\left\|\mathrm{dev}\;\sigma \right\|$ defined by Equation (8). This shows that the function $\;\sigma_{eff}\left(\sigma \right)$ is isotropic. The function polar to γ(σ) has the form:

(17) $$\eta \to \gamma^{o}\left(\eta \right)=\sqrt{2{\left(\mathrm{Tr}\;\eta \right)}^{2}+\frac{2}{3}{\left\|\mathrm{dev}\;\eta \right\|}^{2}}.$$

Thus, we see that its construction can be performed by inverting the coefficients in Equation (16). The simplicity of this construction follows from the orthogonality of the tensors Tr  σ⋅I and dev  σ and from Equation (9). Thus, the yield condition has the form of Equation (13) and $F(\;\sigma )=\gamma \left(\sigma \right)-\sigma_{0}$. It is seen that in the considered case of the plane stress, the function γ(σ) has all the properties of a norm; in particular, it vanishes only if all stress components vanish.

**Remark** **1.** *In the plane strain problem of structures made of the materials satisfying Equation (14), the function γ(σ)* *does not have the properties of a norm, since the condition γ(σ)=0* *implies σ=αI, α∈R. Thus, within the theory of elasto-plasticity, there is a vital difference between the plane stress and plane strain cases. The results of the present paper cannot be transferred to the plane strain case; it would require an independent analysis.*

The elastic energy stored in the plate, expressed in terms of the virtual stress field **τ**, is given by:

(18) $$W(\tau )=\frac{1}{2}\int_{\Omega }\left[\frac{1}{2k}{\left(\mathrm{Tr}\;\tau \right)}^{2}+\frac{1}{2\mu }{\left\|\mathrm{dev}\;\tau \right\|}^{2}\right]dxdy,\;$$

the operations Tr(·) and dev(·) are defined by Equation (10) for *d* = 2 and Equation (6). The bulk modulus k(x,y) and the shear modulus μ(x,y) are determined like in the classical theory of in-plane loaded plates; their units are N/m.

Any virtual stress field **τ** must satisfy the equilibrium equations, both local and along the loaded boundary of the domain, hence it should satisfy the virtual work equation:

(19) $$\int_{\Omega }\tau \cdot \varepsilon (v)dxdy=f(v)\;\;\forall v\in V(\Omega ),$$

where ε(v) is given by Equation (11), while *f*(**v**) represents the virtual work of loads. If the body forces are neglected and the tractions of intensity g are applied along the part $\Gamma_{1}$ of the contour ∂Ω, then:

(20) $$f(v)=\int_{\Gamma_{1}}g\cdot vds,$$

where *s* is the natural parameter of the contour $\Gamma_{1}$. The variational equation implies:

— the local equations of equilibrium

(21) $$\frac{\partial \tau_{x}}{\partial x}+\frac{\partial \tau_{xy}}{\partial y}=0,\;\;\frac{\partial \tau_{yx}}{\partial x}+\frac{\partial \tau_{y}}{\partial y}=0,\;\;\tau_{xy}=\tau_{yx}$$

— the static boundary conditions

(22) $$\tau_{x}n_{x}+\tau_{yx}n_{y}=g_{x},\;\;\tau_{xy}n_{x}+\tau_{y}n_{y}=g_{y}$$

on the contour $\Gamma_{1}$. Such statically admissible stress fields form the set Σ(Ω). In the problem considered, the stresses undergo the plasticity condition:

(23) $$\gamma \left(\tau \left(x,y\right)\right)\leq \sigma_{0}\;\mathrm{for}\;\mathrm{points}\;(x,y)\;\mathrm{within}\;\Omega$$

where γ(τ) is given by Equation (16). The set of stress fields satisfying Equation (23) will be denoted by K(Ω). Thus, τ∈Σ(Ω)∩K(Ω). According to the results [15] concerning the Hencky-Nadai-Ilyushin theory, the unknown stress field σ is the minimizer of the problem:

(24) $$J(k,\mu )=\min\;\left\{\int_{\Omega }\left[\frac{1}{k}{\left(\mathrm{Tr}\;\tau \right)}^{2}+\frac{1}{\mu }{\left\|\mathrm{dev}\;\tau \right\|}^{2}\right]dxdy|\;\mathrm{such}\;\mathrm{that}\;\tau \in \Sigma \left(\Omega \right),\tau \in K\left(\Omega \right)\right\}.$$

The stress field **σ** is accompanied with the displacement fields $u_{x},u_{y}$ such that:

(25) $$\begin{array}{l} \frac{\partial u_{x}}{\partial x}=\frac{k+\mu }{4k\mu }\sigma_{x}-\frac{k-\mu }{4k\mu }\sigma_{y}+\varepsilon_{x}^{p}(x,y),\\ \frac{\partial u_{y}}{\partial y}=-\frac{k-\mu }{4k\mu }\sigma_{x}+\frac{k+\mu }{4k\mu }\sigma_{y}+\varepsilon_{y}^{p}(x,y),\\ \frac{1}{2}\left(\frac{\partial u_{x}}{\partial y}+\frac{\partial u_{y}}{\partial x}\right)=\frac{1}{2\mu }\sigma_{xy}+\varepsilon_{xy}^{p}(x,y) \end{array}$$

where the components of the so-called plastic strains $\varepsilon_{x}^{p}(x,y),\;\varepsilon_{y}^{p}(x,y),\;\varepsilon_{xy}^{p}(x,y)$ are not kinematically compatible; they are not associated with any displacement field, i.e., there does not exist a vector field **v** such that Equation (11) holds. The pair $\left(\sigma ,\varepsilon^{p}\right)$ satisfies the variational inequality:

(26) $$\sigma \cdot \varepsilon^{p}\geq \tau \cdot \varepsilon^{p}\;\;\forall \tau \;\mathrm{such}\;\mathrm{that}\;\gamma \left(\tau \right)\leq \sigma_{0}$$

representing the celebrated Hill’s principle of maximal plastic work. Having Equation (25), the equilibrium Equations (21) and (22) and the plasticity condition Equation (23) can construct the displacement fields $u_{x},u_{y}$ in the elasto-plastic structure. 

While the plasticity condition cancels extrema of the stress fields (see [22]), admitting the plastic components of strain degenerates the layout of the displacement fields (see [23]). 

Let us emphasize once again that the considered 2D problem is viewed as the plane stress problem of statics of a plate of constant thickness *b*. Thus, the intensities of the tractions, the elastic moduli, the plastic limit $\sigma_{0}$ as well as the stress components are measured in N/m. The virtual work and the compliance have the units Nm. 

## 3. The Isotropic Material Design (IMD) Method within the Elasto-Plastic Range

The aim is to construct the strongest transversely homogeneous plate made of the isotropic material of non-negative bulk and shear moduli; just these moduli are the only design variables of the problem. The unit cost of the design is assumed as trace of the Hooke tensor. In the 2D case, the eigenvalues of the Hooke tensor are: 2k,2μ,2μ, hence the unit cost is equal to 2k+4μ. The total cost is bounded by a constant $\Lambda_{0}$:

(27) $$\int_{\Omega }\left(2k+4\mu \right)dxdy\leq \Lambda_{0}.$$

We shall assume in the sequel that the permissible stress $\sigma_{0}$ does not depend on the design variables (k,μ). Thus, the optimum design problem assumes the form:

(28) $$Y=\min\left\{J(k,\mu )|k\geq 0,\mu \geq 0,\int_{\Omega }\left(2k+4\mu \right)dxdy\leq \Lambda_{0}\right\},$$

*Y* being the compliance of the optimal structure. Let us insert Equation (24) and perform minimization over the design variables (k,μ), by making use of the sets Σ(Ω),K(Ω) being independent of the design variables. The operation of minimization over the design variables can be performed by using the minimization result (see [24]):

(29) $$\begin{array}{cc} \min & \left\{\int_{\Omega }\left(\frac{a_{1}}{u_{1}}+\frac{a_{2}}{u_{2}}\right)dxdy|\;\mathrm{over}\;u_{1},\;u_{2}\;\mathrm{such}\;\mathrm{that}:\;u_{1}\geq u_{2}\geq 0,\;\int_{\Omega }(u_{1}+u_{2})dx\leq \Lambda_{0}\right\}\\ & =\frac{1}{\Lambda_{0}}{\left(\int_{\Omega }\left(\sqrt{a_{1}}+\sqrt{a_{2}}\right)dxdy\;\right)}^{2} \end{array}$$

in which $a_{1}\geq 0,a_{2}\geq 0$ are given functions in the domain Ω. The equality above is attained for:

(30) $${\hat{u}}_{i}\left(x,y\right)=\frac{\Lambda_{0}}{\int_{\Omega }\left(\sqrt{a_{1}}+\sqrt{a_{2}}\right)dxdy}\sqrt{a_{i}(x,y)}\;,\;i\;=1,\;2.$$

Upon assuming:

(31) $$a_{1}=2{\left(\mathrm{Tr}\;\tau \right)}^{2},\;a_{2}=4{\left\|\mathrm{dev}\;\tau \right\|}^{2},\;u_{1}=2k,\;u_{2}=4\mu$$

we find:

(32) $$Y=\frac{1}{\Lambda_{0}}\Pi_{}^{2},$$

where:

(33) $$\Pi =\min\left\{\int_{\Omega }\rho \left(\tau \right)\;dxdy\;|\tau \in \Sigma \left(\Omega \right)\cap K\left(\Omega \right)\right\}\;(P)\;$$

and the integrand is expressed by the norm:

(34) $$\rho \left(\tau \right)=\left|\mathrm{Tr}\;\tau \right|+\sqrt{2}\left\|\mathrm{dev}\;\tau \right\|.\;$$

Assume that the problem (*P*) is solvable upon appropriate mathematical modification; let $\tau^{*}$ be the minimizer. The optimal moduli are expressed by:

(35) $$k^{*}(x,y)=E_{0}\frac{1}{2}\frac{\left|\mathrm{Tr}\;\tau^{*}(x,y)\right|}{\Pi /\left|\Omega \right|},\;\;\mu^{*}(x,y)=E_{0}\frac{\sqrt{2}}{4}\frac{\left\|\mathrm{dev}\;\tau^{*}(x,y)\right\|}{\Pi /\left|\Omega \right|},$$

where $E_{0}=\Lambda_{0}/\left|\Omega \right|$. It is easy to note that Equation (27) is satisfied sharply. 

One can prove that the stress field in the optimal plate (in which the elastic moduli are determined by Equation (35)) coincides with the stress field $\tau^{*}$ solving the problem (*P*). Thus, the method put forward makes it possible to form the safely designed least compliant plate structure in which the stress field satisfies both the equilibrium equations and the yield stress condition (Equation (23)).

## 4. The IMD Design: The Displacement-Based Elasto-Plastic Formulation

### 4.1. The General Form of the Problem

The IMD method requires the construction of the problem dual to (*P*) (see Equation (33)). To this end, we first release the constraints τ∈Σ(Ω), and by treating the virtual displacements in Equation (19) as Lagrange multipliers, we rearrange (*P*) to the form:

(36) $$\Pi =\underset{\tau \in K\left(\Omega \right)}{\min}\underset{v\in V(\Omega )}{\max}\left\{f(v)+\int_{\Omega }[\rho \left(\tau \right)-\tau \cdot \varepsilon \left(v\right)]dxdy\;\right\}.\;$$

The operations: min and max can be interchanged (see [25]), which makes it possible to re-write Equation (36) as below:

(37) $$\begin{array}{l} \Pi =\underset{v\in V(\Omega )}{\max}\left\{f(v)+\underset{\tau \in K\left(\Omega \right)}{\min}\int_{\Omega }[\rho \left(\tau \right)-\tau \cdot \varepsilon \left(v\right)]dxdy\;\right\}\\ =\underset{v\in V(\Omega )}{\max}\left\{f(v)-\underset{\tau \in K\left(\Omega \right)}{\max}\int_{\Omega }[\tau \cdot \varepsilon \left(v\right)-\rho \left(\tau \right)]dxdy\;\right\}\\ =\underset{v\in V(\Omega )}{\max}\left\{f(v)-\sigma_{0}\int_{\Omega }h(\varepsilon (v))dxdy\;\right\} \end{array}$$

where

(38) $$h(\varepsilon )=\frac{1}{\sigma_{0}}\underset{\begin{array}{l} \tau \in E_{s}^{2}\\ \gamma \left(\tau \right)\leq \sigma_{0} \end{array}}{\max}\left(\tau \cdot \varepsilon -\rho \left(\tau \right)\right).\;$$

In the next step, we shall find the explicit form of Equation (38); its form will not involve the parameter $\sigma_{0}$.

### 4.2. Construction of the Potential h(ε) and the Explicit Formulation of the Problem Dual to (P)

By using Equation (9) for the scalar product of two tensors from $E_{s}^{2}$, taking into account Equations (34) and (16) and remembering that *d* = 2, we rewrite the local problem (Equation (38)) in the form:

(39) $$\sigma_{0}h(\varepsilon )=\underset{\begin{array}{c} \tau \in E_{s}^{2}\\ {\left(\mathrm{Tr}\;\tau \right)}^{2}+3{\left\|\mathrm{dev}\;\tau \right\|}^{2}\leq 2{\left(\sigma_{0}\right)}^{2} \end{array}}{\max}\left\{\mathrm{Tr}\;\tau \cdot \mathrm{Tr}\;\varepsilon +\mathrm{dev}\;\tau \cdot \mathrm{dev}\;\varepsilon -\left|\mathrm{Tr}\;\tau \right|-\sqrt{2}\left\|\mathrm{dev}\;\tau \right\|\right\}.$$

Let us introduce the notation:

(40) $$a=\mathrm{Tr}\;\varepsilon ,\;p=\mathrm{Tr}\;\tau ,\;c=\frac{1}{2}\left(\varepsilon_{x}-\varepsilon_{y}\right),\;b=\sqrt{2}\varepsilon_{xy},\;q=\frac{1}{2}\left(\tau_{x}-\tau_{y}\right),\;r=\sqrt{2}\tau_{xy}$$

and re-write Equation (39) in the form $\sigma_{0}h\left(\varepsilon \right)=\sigma_{0}h_{1}\left(a,c,b\right)$, where:

(41) $$\sigma_{0}h_{1}\left(a,c,b\right)=\underset{\begin{array}{c} p,q,r\in R\\ p^{2}+3\left(2q^{2}+r^{2}\right)\leq 2{\left(\sigma_{0}\right)}^{2} \end{array}}{\max}\left\{ap-\left|p\right|+2qc+rb-\sqrt{2}\sqrt{2q^{2}+r^{2}}\right\}.\;$$

Let us introduce a new notation:

(42) $$x=\frac{p}{\sqrt{2}\sigma_{0}},\;y=\frac{\sqrt{3}q}{\sigma_{0}},\;z=\sqrt{\frac{3}{2}}\frac{r}{\sigma_{0}},\;b_{1}=b/\sqrt{2}.\;$$

Equation (41) simplifies to the form:

(43) $$\sigma_{0}h_{1}\left(a,c,b\right)=\underset{\;(x,y,z)\in B(0,1)\;}{\max}\left\{\sqrt{2}\left(ax-\left|x\right|\right)+\frac{2}{\sqrt{3}}\left(cy+b_{1}z-\sqrt{y^{2}+z^{2}}\right)\right\}\sigma_{0}$$

where *B*(0,1) is a unit ball: $x^{2}+y^{2}+z^{2}\leq 1$. We see that the parameter $\sigma_{0}$ is cancelled. Now, we introduce the spherical parameterization:

(44) x=tcosϑ, y=tsinϑcosφ, z=tsinϑsinφ, 0≤ϑ≤π, 0≤φ≤2π, |t|≤1.

Operation max over φ gives:

(45) $$\cos\varphi =\frac{c}{\sqrt{c^{2}+{\left(b_{1}\right)}^{2}}},\;\sin\varphi =\frac{b_{1}}{\sqrt{c^{2}+{\left(b_{1}\right)}^{2}}},\;$$

which simplifies Equation (43) to the form:

(46) $$h_{1}=\underset{\begin{array}{c} \left|t\right|\leq 1\\ \;0\leq \vartheta \leq \;\pi \end{array}}{\max}t\left\{\sqrt{2}\left(a\cos\vartheta -\left|\cos\vartheta \right|\right)+\frac{2}{\sqrt{3}}\left(\eta -1\right)\sin\vartheta \right\}$$

where:

(47) $$a=\frac{1}{\sqrt{2}}\left(\varepsilon_{x}+\varepsilon_{y}\right),\;\eta =\sqrt{c^{2}+{\left(b_{1}\right)}^{2}}\;\mathrm{or}\;\eta =\frac{1}{\sqrt{2}}\left\|\mathrm{dev}\;\varepsilon \right\|=\sqrt{\frac{1}{4}{\left(\varepsilon_{x}-\varepsilon_{y}\right)}^{2}+{\left(\varepsilon_{xy}\right)}^{2}}$$

Let us introduce a division of the set $E_{s}^{2}$ into the subdomains:

(48) $$\begin{array}{l} D_{0}=\left\{\varepsilon \in E_{s}^{2}|\left|\mathrm{Tr}\;\varepsilon \right|\leq 1,\;\left\|\mathrm{dev}\;\varepsilon \right\|\leq \sqrt{2}\right\},D_{1}^{\prime }=\left\{\varepsilon \in E_{s}^{2}|\mathrm{Tr}\;\varepsilon \geq 1,\;\left\|\mathrm{dev}\;\varepsilon \right\|\leq \sqrt{2}\right\}\\ D_{1}^{\prime \prime }=\left\{\varepsilon \in E_{s}^{2}|\mathrm{Tr}\;\varepsilon \leq -1,\;\left\|\mathrm{dev}\;\varepsilon \right\|\leq \sqrt{2}\right\},D_{2}^{\prime }=\left\{\varepsilon \in E_{s}^{2}|\mathrm{Tr}\;\varepsilon \geq 1,\;\left\|\mathrm{dev}\;\varepsilon \right\|\geq \sqrt{2}\right\}\\ D_{2}^{\prime \prime }=\left\{\varepsilon \in E_{s}^{2}|\mathrm{Tr}\;\varepsilon \leq -1,\;\left\|\mathrm{dev}\;\varepsilon \right\|\geq \sqrt{2}\right\},D_{3}=\left\{\varepsilon \in E_{s}^{2}|\left|\mathrm{Tr}\;\varepsilon \right|\leq 1,\;\left\|\mathrm{dev}\;\varepsilon \right\|\geq \sqrt{2}\right\} \end{array}$$

**Remark** **2.** *The set* $D_{0}$ *coincides with the set:*

(49) $$\rho^{o}\left(\varepsilon \right)\leq 1$$

*where $\rho^{o}\left(\cdot \right)$* *is the function polar to ρ(⋅). Indeed, the function $\rho^{o}\left(\cdot \right)$* *has the form:*

(50) $$\rho^{o}\left(\varepsilon \right)=\max\left\{\left|\mathrm{Tr}\;\varepsilon \right|,\frac{1}{\sqrt{2}}\left\|\mathrm{dev}\;\varepsilon \right\|\right\}$$

*derived in [14], which confirms the above observation.*

The division (Equation (48)) of $E_{s}^{2}$ into subdomains can be shown in the plane of principal strains. Let us recall that:

(51) $$\mathrm{Tr}\;\varepsilon =\frac{1}{\sqrt{2}}\left(\varepsilon_{I}+\varepsilon_{II}\right),\;\left\|\mathrm{dev}\;\varepsilon \right\|=\frac{1}{\sqrt{2}}\left|\varepsilon_{I}-\varepsilon_{II}\right|$$

**Figure 1:**
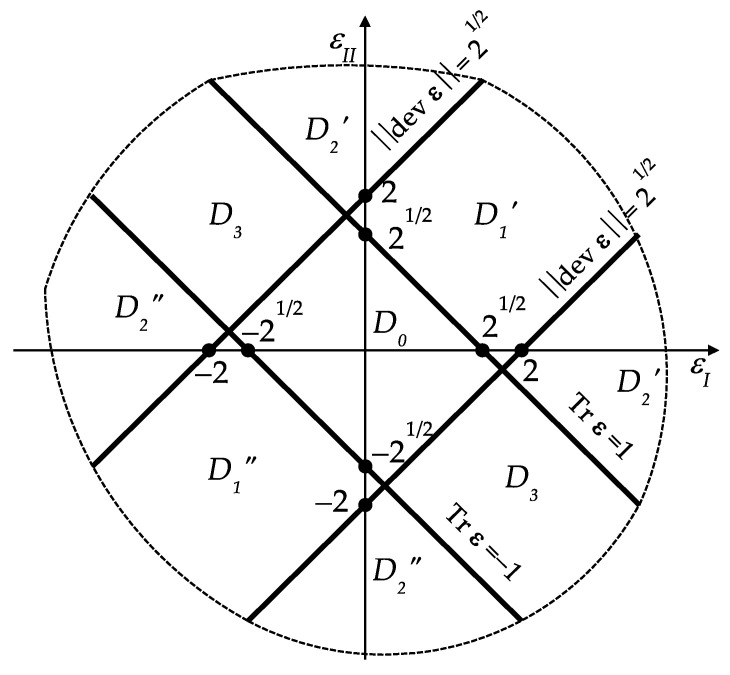
The division (Equation (48)) of the plane of principal strains into subdomains.

**Figure 2:**
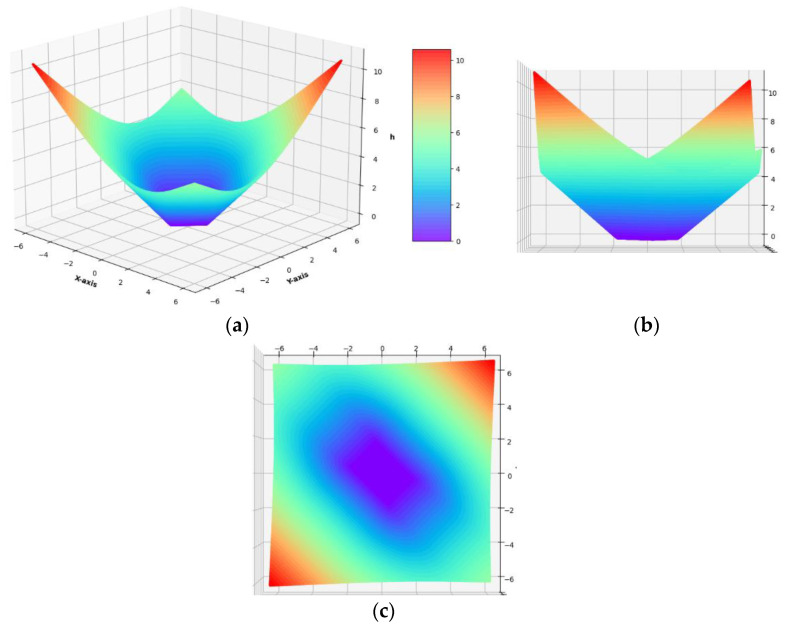
Scatter plots of the function *h*(**ε**) in the plane of principal strains ε_I_, ε_II_ denoted as *x*-axis, *y*-axis, respectively. The axonometric (**a**), side (**b**) and top (**c**) view, respectively.

Now, we are ready to show the explicit formula for the potential h(ε) defined by Equation (38), see Figure 1 and Figure 2.

(52) $$h(\varepsilon )=\left\{ \begin{array}{l} \begin{array}{c} \sqrt{2}\left|\mathrm{Tr}\;\varepsilon -1\right|\;\varepsilon \in D_{1}^{\prime }\\ 0\;\;\;\;\;\;\varepsilon \in D_{0}\\ \sqrt{2}\left|\mathrm{Tr}\;\varepsilon +1\right|\;\varepsilon \in D_{1}^{\prime \prime } \end{array}\\ \begin{array}{c} \sqrt{\frac{2}{3}}\sqrt{3{\left(\mathrm{Tr}\;\varepsilon -1\right)}^{2}+{\left(\left\|\mathrm{dev}\;\varepsilon \right\|-\sqrt{2}\right)}^{2}}\;\varepsilon \in D_{2}^{\prime }\\ \sqrt{\frac{2}{3}}\;\left(\left\|\mathrm{dev}\;\varepsilon \right\|-\sqrt{2}\right)\;\;\;\;\;\;\;\;\;\varepsilon \in D_{3}\\ \sqrt{\frac{2}{3}}\sqrt{3{\left(\mathrm{Tr}\;\varepsilon +1\right)}^{2}+{\left(\left\|\mathrm{dev}\;\varepsilon \right\|-\sqrt{2}\right)}^{2}}\;\varepsilon \in D_{2}^{\prime \prime } \end{array} \end{array}\right.$$

The function h(ε) is continuous, i.e., it is continuously stitched along the lines $\mathrm{Tr}\;\varepsilon =1,\;\mathrm{Tr}\;\varepsilon =-1,\left\|\mathrm{dev}\;\varepsilon \right\|=\sqrt{2}$ (see Figure 1). Moreover, it is convex, of linear growth outside the central domain *D*_0_, vanishes at ε=0 and is non-negative. In conclusion, the problem dual to (*P*) (see Equation (33)) assumes the form:

(53) $$\Pi =\underset{v\in V(\Omega )}{\max}\left\{f(v)-\sigma_{0}\int_{\Omega }h(\varepsilon (v))dxdy\;\right\}.\;(P^{*})$$

One can prove that this value coincides with Equation (33), and the duality gap is zero. The pair (*P*), (P*) constitutes the LCP problem in the meaning of Bouchitté and Fragalà [13].

**Remark** **3.** *Within the linear elastic range, Equation (53) reduces to:*

(54) $$\Pi =\underset{v\in V(\Omega )}{\max}\left\{f(v)|\rho^{o}\left(\varepsilon (v\left(x,y\right))\right)\leq 1\;a.e.\;\mathrm{in}\;\Omega \right\}.\;$$

*Note that the locking domain $\rho^{o}\left(\varepsilon (v\left(x,y\right))\right)\leq 1$* *is just the domain D_0_ given by Equation (48) (see Remark 2). Moreover, one can prove that the displacement field $\left(u_{x},u_{y}\right)$* *in the optimal structure (whose moduli are given by Equation (35)) is proportional to the maximizer $v^{*}$* *of Equation (53):*

(55) $$u=\frac{\Pi }{\Lambda_{0}}v^{*}.\;$$

*Thus, the optimization process introduces the bounds on strains, while the values of stresses follow the values of the optimal elastic moduli.*

## 5. Construction of the Approximants of Statically Admissible Stresses

The optimal moduli $k^{*},\mu^{*}$ are determined by the solution to Equation (33). Therefore, it is thought appropriate to concentrate attention just on this problem and not on its dual form (Equation (53)). The aim of the present section is to show the numerical construction of sequences of sets $\Sigma_{h}\left(\Omega \right)$ approximating the set Σ(Ω) of statically admissible stresses, e.g., stresses equilibrating the given boundary traction load, hence satisfying the equilibrium Equations (21) and (22); index *h* symbolizes the mesh density parameter.

The description of the sequence of approximating sets $\Sigma_{h}\left(\Omega \right)$ needs specific notation, linked directly with the C++ programming syntax. The reader is asked to accept that the indices will start now from 0, not from 1. In particular, from now onward, the axes (*x*,*y*) will be denoted by $\left(x_{0},x_{1}\right)$; consequently, we shall write $f_{0},f_{1}$ instead of $f_{x},f_{y}$ and $f_{00},f_{01},f_{10},f_{11}$ instead of $f_{x},f_{xy},f_{yx},f_{y}$. 

If Ω is a polygon, then the stress-based finite element method can be formulated as: find the interpolation $\sigma_{h}\in \Sigma_{h}\subset \Sigma \left(\Omega \right)$ of the statically admissible stress tensor field σ∈Σ(Ω), such that:

(56) $$\forall \upsilon_{h}\in V_{h}\;\int_{\Omega }\sigma_{h}\cdot D\upsilon_{h}\;d\Omega =\int_{\Gamma_{1}}g\cdot \upsilon_{h}\;ds,\;$$

where *Dv* represents the gradient of a vector field *v* and $V_{h}\subset V\left(\Omega \right)$ is the finite element-wise subspace of functions $\upsilon_{h}=\left(\upsilon_{h0},\upsilon_{h1}\right):\Omega \to R^{2}$ spanned by the polynomials of an appropriate degree. The P_1_ (or Q_1_) degree polynomials *p* = *p*(**x**)

$$\forall x={\left[\begin{array}{cc} x_{0} & x_{1} \end{array}\right]}^{T}\in R^{2}\;p=p\left(x\right)=p_{00}+p_{10}x_{0}+p_{01}x_{1}\left(+p_{11}x_{0}x_{1}\right),\;p_{00},p_{10},p_{01}\left(,p_{11}\right)\in R$$

are adopted in this paper (see [26]).

**Figure 3:**
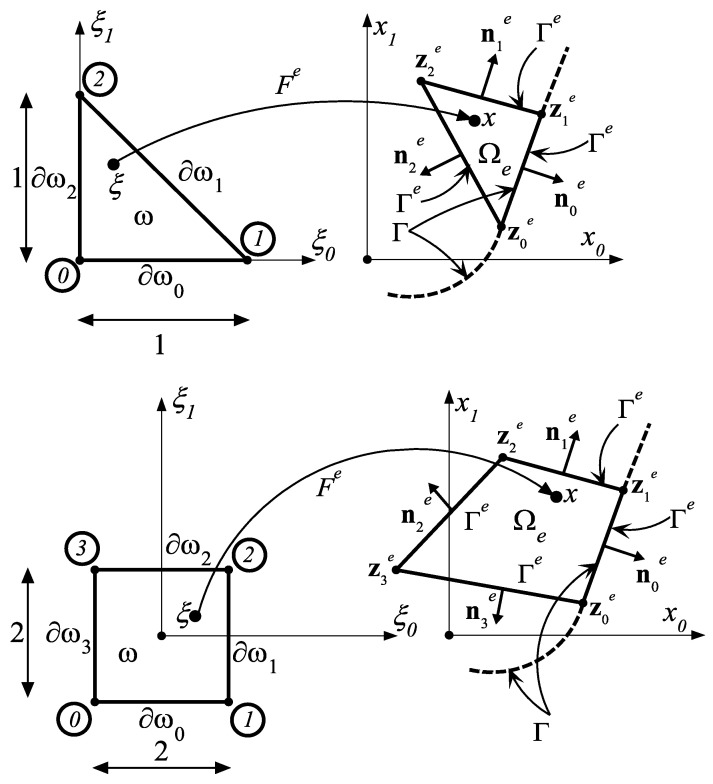
The mapping **F***^e^* defining the relation between the triangular and quadratic master element ω (on the left) and arbitrary current finite element Ω*_e_* (on the right).

The finite element mesh in the domain Ω is composed of *M* 3- (or 4-node) finite elements $\Omega_{e}\subset \Omega$ covering the whole domain, provided it is a polygon. Let $\upsilon_{h}^{e}={\upsilon_{h}|}_{\Omega^{e}}$ be the truncation of $\upsilon_{h}$ to the *e*-th element. Thus, the values $\upsilon_{h}\left(x\right)=\left(\upsilon_{h0}\left(x\right),\upsilon_{h1}\left(x\right)\right)$ of the vector field $\upsilon_{h}=\left(\upsilon_{h0},\upsilon_{h1}\right)\in V_{h}$ truncated to the *e*-th element may be equivalently represented as two-dimensional vector:

(57) $$\upsilon_{h}^{e}\left(x\right)={\left[\begin{array}{cc} \upsilon_{h0}^{e}\left(x\right) & \upsilon_{h1}^{e}\left(\xi \right) \end{array}\right]}^{T}={\left[\begin{array}{cc} \sum_{i=0}^{m}\upsilon_{2i}^{e}\;\varphi_{i}^{e}\left(x\right) & \sum_{i=0}^{m}\upsilon_{2i+1}^{e}\;\varphi_{i}^{e}\left(x\right) \end{array}\right]}^{T}\in R^{2},\;m=2\;\left(3\right),x\in \Omega_{e},$$

where $\upsilon_{0}^{e},\upsilon_{1}^{e},\ldots ,\upsilon_{2m}^{e},\upsilon_{2m+1}^{e}$ are the unknown values of the scalar functions $\upsilon_{hi}^{e}\left(\cdot \right)$, *i* = 0, 1 at three (or four) subsequent vertices of the triangle (quadrilateral) $\Omega_{e}$, while the polynomials

$$\varphi_{i}^{e}:\Omega_{e}\to R$$,

*i* = 0, 1, …, *m* are the shape functions, which depend explicitly on the Cartesian co-ordinates $z_{i}^{e}=\left(z_{i0}^{e},z_{i1}^{e}\right)\in R^{2}$, *i* = 0, 1, …, *m* of the three (*m* = 2) or four (*m* = 3) vertices defining a triangular or quadrilateral finite element Ω*_e_* (see Figure 3). In the case considered, the formulae defining the shape functions in Equation (57) are relatively simple. However, even here it is thought appropriate to avoid using the functions $\varphi_{i}^{e}\left(\cdot \right)$ in Equation (57) and replace them by far more simple shape functions: $\phi_{i}:\omega \to R,\;i=0,1,\ldots ,m$, defined on the master element; in our problem, these functions are expressed by:

(58) $$\phi_{0}\left(\xi \right)=1-\xi_{0}-\xi_{1},\;\phi_{1}\left(\xi \right)=\xi_{0},\;\phi_{2}\left(\xi \right)=\xi_{1}$$

for triangular reference (master) element and

(59) $$\begin{array}{l} \phi_{0}\left(\xi \right)=\frac{1}{4}\left(1-\xi_{0}-\xi_{1}+\xi_{0}\xi_{1}\right),\;\phi_{1}\left(\xi \right)=\frac{1}{4}\left(1+\xi_{0}-\xi_{1}-\xi_{0}\xi_{1}\right)\\ \phi_{2}\left(\xi \right)=\frac{1}{4}\left(1+\xi_{0}+\xi_{1}+\xi_{0}\xi_{1}\right),\;\phi_{3}\left(\xi \right)=\frac{1}{4}\left(1-\xi_{0}+\xi_{1}-\xi_{0}\xi_{1}\right) \end{array}$$

for square master element, where $\xi ={\left[\begin{array}{cc} \xi_{0} & \xi_{1} \end{array}\right]}^{T}\in \omega \subset R^{2}$. The implementation of the shape functions (Equations (58) and (59)) for an arbitrary $\Omega_{e}$ element necessitates the introduction of a family of mappings $F^{e}=\left(F_{0}^{e},F_{1}^{e}\right):\omega \to \Omega_{e}\;,\;F^{e}\left(\omega \right)=\Omega_{e}$, which link the master element ω with an arbitrary element $\Omega_{e}$ such that $\varphi_{i}^{e}\left(x\right)=\phi_{i}\left(\xi \right),\;x=F^{e}\left(\xi \right)\in \Omega_{e},\;\xi \in \omega$. This makes it possible to replace Equation (57) with a much simpler one:

(60) $$\;\upsilon_{h}^{e}\left(x\right)={\left[\begin{array}{cc} \upsilon_{h0}^{e}\left(x\right) & \upsilon_{h1}^{e}\left(x\right) \end{array}\right]}^{T}={\left[\begin{array}{cc} \sum_{i=0}^{m}\upsilon_{2i}^{e}\;\phi_{i}\left(\xi \right) & \sum_{i=0}^{m}\upsilon_{2i+1}^{e}\;\phi_{i}\left(\xi \right) \end{array}\right]}^{T},\;x=F^{e}\left(\xi \right),\;\xi \in \omega .$$

The geometric mapping $F^{e}:\omega \to \Omega_{e}$ is defined in a similar manner as the field $\upsilon_{h}^{e}$ has been constructed. Using the shape functions $\phi_{i}$ and the Cartesian coordinates of nodes $z_{i}^{e}={\left[\begin{array}{cc} z_{i0}^{e} & z_{i1}^{e} \end{array}\right]}^{T}$ of the finite element $\Omega_{e}$, we have the following simple relation:

(61) $$\forall \xi =\left(\xi_{0},\xi_{1}\right)\in \omega \subset R^{2}\;F^{e}\left(\xi \right)=\left(F_{0}^{e}\left(\xi \right),F_{1}^{e}\left(\xi \right)\right)=\sum_{i=0}^{m}z_{i}^{e}\;\phi_{i}\left(\xi \right)\in \Omega_{e}\subset R^{2}$$

The derivative of this mapping is a linear operator represented by the matrix:

(62) $$DF^{e}\left(\xi \right)=\left[\begin{array}{cc} \frac{\partial F_{0}^{e}}{\partial x_{0}}\left(\xi \right) & \frac{\partial F_{0}^{e}}{\partial x_{1}}\left(\xi \right)\\ \frac{\partial F_{1}^{e}}{\partial x_{0}}\left(\xi \right) & \frac{\partial F_{1}^{e}}{\partial x_{1}}\left(\xi \right) \end{array}\right]$$

defined on master element ω (constant only for triangular element). On the basis of the easily calculated gradients $\nabla \phi_{i}\left(\xi \right)$ of the shape functions $\phi_{i}\left(\xi \right),\;\xi \in \omega$, the gradients $\nabla \varphi_{i}^{e}\left(x\right)$, *i* = 0, 1, …, *m* of the shape functions $\varphi_{i}^{e}\left(x\right),\;x=F^{e}\left(\xi \right)\in \Omega_{e}$ are computed by:

(63) $$\nabla \varphi_{i}^{e}\left(x\right)=\nabla \varphi_{i}^{e}\left(F^{e}\left(\xi \right)\right)={\left(DF^{e}\left(\xi \right)\right)}^{-T}\nabla \phi_{i}\left(\xi \right)$$

drawing upon the knowledge of the matrix ${\left(DF^{e}\left(\xi \right)\right)}^{-T}$ being inverse-transpose to the matrix represented in Equation (62). 

In the adopted, purely stress approach, it is assumed that the interpolation of all stress components is defined analogously to the interpolation of the test displacement components $\upsilon_{h0}^{e}\left(\cdot \right),\;\upsilon_{h1}^{e}\left(\cdot \right)$, i.e.:

(64) $$\begin{array}{l} {\bar{\sigma }}_{h}^{e}\left(x\right)={\left[\begin{array}{ccc} \sigma_{h00}^{e}\left(x\right) & \sigma_{h11}^{e}\left(x\right) & \sigma_{h01}^{e}\left(x\right) \end{array}\right]}^{T}={\left[\begin{array}{ccc} \sum_{i=0}^{m}\tau_{3i}^{e}\;\phi_{i}\left(\xi \right) & \sum_{i=0}^{m}\tau_{3i+1}^{e}\;\phi_{i}\left(\xi \right) & \sum_{i=0}^{m}\tau_{3i+2}^{e}\;\phi_{i}\left(\xi \right) \end{array}\right]}^{T},\\ \sigma_{h10}^{e}\left(x\right)=\sigma_{h01}^{e}\left(x\right),\;x=F^{e}\left(\xi \right),\xi \in \omega \end{array}$$

where $\tau_{0}^{e},\tau_{1}^{e},\tau_{2}^{e},\ldots ,\tau_{3m+0}^{e},\tau_{3m+1}^{e},\tau_{3m+2}^{e}$ are the unknown values of the scalar functions $\sigma_{h00}^{e}\left(\cdot \right),\sigma_{h11}^{e}\left(\cdot \right),\sigma_{h01}^{e}\left(\cdot \right)$ (i.e., nodal stresses) at three (or four) subsequent vertices of the triangle (or quadrilateral) $\Omega_{e}$.

**Figure 4:**
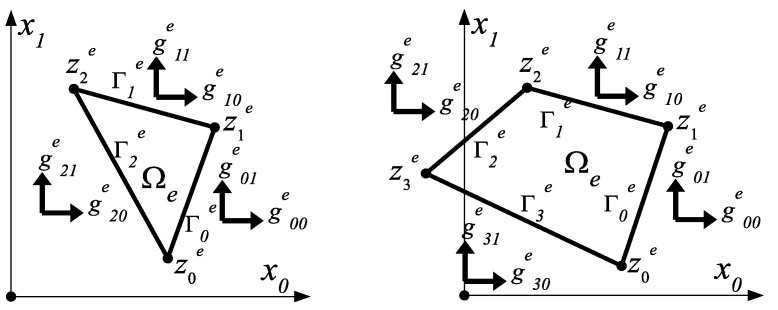
Notation of traction forces applied to all three (or four) edges $\Gamma_{i}^{e}\;i=0,1,2\left(,3\right)$ of finite elements.

For the sake of simplicity, we assume that the load **g** applied to the boundary $\Gamma_{1}\subset \Gamma =\partial \Omega$ of the design domain may have a different but constant value on selected sides of the polygon Ω, i.e.,$\forall x\in \Gamma \;g\left(x\right)=g={\left[\begin{array}{cc} g_{0} & g_{1} \end{array}\right]}^{T}=const$, that is, we assume that a constant load is applied to the edge of any finite element, which is a fragment of the edge of the design domain Ω, possibly changing its value depending on the *e*-th number of the finite element $\Omega_{e}$. This allows us to assume that the vector **g** can be defined by three or four constants on each edge vector (see Figure 4):

(65) $$g_{i}^{e}=\left[\begin{array}{c} g_{i0}^{e}\\ g_{i1}^{e} \end{array}\right]\in R^{2},\;i\;=\;0,\;1,\;\ldots ,\;m.$$

The calculation of the integral over the entire domain Ω and its boundary Γ (strictly Γ_1_) in the variational Equation (56) can be reduced (as in classical, displacement-based FEM) to the calculation of the sum of the integrals over finite elements $\Omega_{e}$ and their selected (i.e., loaded) boundaries $\Gamma_{e^{\prime }}$, which coincide with the boundary $\Gamma_{1}\subset \Gamma \subset \partial \Omega$:

(66) $$\forall \upsilon_{h}\in V_{h}\;\sum_{e}\int_{\Omega_{e}}\sigma_{h}^{e}\cdot D\upsilon_{h}^{e}\;d\Omega_{e}=\sum_{e^{\prime }}\int_{\Gamma_{e^{\prime }}}g^{e^{\prime }}\cdot \upsilon_{h}^{e^{\prime }}ds$$

Integration over $\Omega_{e}$ and $\Gamma_{e^{\prime }}$ is shifted to the reference element ω and its boundaries $\partial \omega_{i}\;,\;i=0,1,\ldots ,m$. The left hand side is computed as follows:

(67) $$\begin{array}{l} \int_{\Omega_{e}}\sigma_{h}^{e}\cdot D\upsilon_{h}^{e}\;dx_{0}dx_{1}=\int_{\omega }\sigma_{h}^{e}\left(F^{e}\left(\xi \right)\right)\cdot D\upsilon_{h}^{e}\left(F^{e}\left(\xi \right)\right)\left|\det DF^{e}\left(\xi \right)\right|\;d\xi_{0}d\xi_{1}=\\ =\int_{\omega }\mathrm{tr}\left(\sigma_{h}^{e}\left(\xi \right)\circ D\upsilon_{h}^{e}\left(\xi \right)\right)\left|\det DF^{e}\left(\xi \right)\right|\;d\xi_{0}\xi_{1}\\ \sigma_{h}^{e}\left(\xi \right)=\left[\begin{array}{cc} \sum_{i=0}^{m}\tau_{3i}^{e}\;\phi_{i}\left(\xi \right) & \sum_{i=0}^{m}\tau_{3i+2}^{e}\;\phi_{i}\left(\xi \right)\\ \sum_{i=0}^{m}\tau_{3i+2}^{e}\;\phi_{i}\left(\xi \right) & \sum_{i=0}^{m}\tau_{3i+1}^{e}\;\phi_{i}\left(\xi \right) \end{array}\right]\;,\;D\upsilon_{h}^{e}\left(\xi \right)=\left[\begin{array}{c} {\left(\nabla \upsilon_{h0}^{e}\left(F^{e}\left(\xi \right)\right)\right)}^{T}\\ {\left(\nabla \upsilon_{h1}^{e}\left(F^{e}\left(\xi \right)\right)\right)}^{T} \end{array}\right]\\ \nabla \upsilon_{h0}^{e}\left(F^{e}\left(\xi \right)\right)=\sum_{i=0}^{m}\upsilon_{2i}^{e}{\left(DF^{e}\left(\xi \right)\right)}^{-T}\nabla \phi_{i}\left(\xi \right)\;,\;\nabla \upsilon_{h1}^{e}\left(F^{e}\left(\xi \right)\right)=\sum_{i=0}^{m}\upsilon_{2i+1}^{e}{\left(DF^{e}\left(\xi \right)\right)}^{-T}\nabla \phi_{i}\left(\xi \right) \end{array}$$

If the triangular element is used, the computation of the right hand side of Equation (56) is performed as below:

(68) $$\begin{array}{l} \int_{\Gamma_{e^{\prime }}}g^{e^{\prime }}\cdot \upsilon_{h}^{e^{\prime }}\;ds=\\ =\int_{0}^{1}g_{0}^{e^{\prime }}\cdot \upsilon_{h}^{e^{\prime }}\left(F^{e^{\prime }}\left(\zeta ,0\right)\right)\sqrt{{\left(DF_{0}^{e^{\prime }}\left(\zeta ,0\right)\right)}^{2}+{\left(DF_{1}^{e^{\prime }}\left(\zeta ,0\right)\right)}^{2}}d\zeta \\ +\int_{0}^{1}g_{1}^{e^{\prime }}\cdot \upsilon_{h}^{e^{\prime }}\left(F^{e^{\prime }}\left(\zeta ,1-\zeta \right)\right)\sqrt{{\left(DF_{0}^{e^{\prime }}\left(\zeta ,1-\zeta \right)\right)}^{2}+{\left(DF_{1}^{e^{\prime }}\left(\zeta ,1-\zeta \right)\right)}^{2}}d\zeta +\\ +\int_{0}^{1}g_{2}^{e^{\prime }}\cdot \upsilon_{h}^{e^{\prime }}\left(F^{e^{\prime }}\left(0,\zeta \right)\right)\sqrt{{\left(DF_{0}^{e^{\prime }}\left(0,\zeta \right)\right)}^{2}+{\left(DF_{1}^{e^{\prime }}\left(0,\zeta \right)\right)}^{2}}d\zeta \end{array}$$

while in the case of quadrilateral elements, the computation is performed in the way:

(69) $$\begin{array}{l} \int_{\Gamma_{e^{\prime }}}g^{e^{\prime }}\cdot \upsilon_{h}^{e^{\prime }}\;ds=\\ =\int_{-1}^{1}g_{0}^{e^{\prime }}\cdot \upsilon_{h}^{e^{\prime }}\left(F^{e^{\prime }}\left(\zeta ,-1\right)\right)\sqrt{{\left(DF_{0}^{e^{\prime }}\left(\zeta ,-1\right)\right)}^{2}+{\left(DF_{1}^{e^{\prime }}\left(\zeta ,-1\right)\right)}^{2}}d\zeta +\\ +\int_{-1}^{1}g_{1}^{e^{\prime }}\cdot \upsilon_{h}^{e^{\prime }}\left(F^{e^{\prime }}\left(1,\zeta \right)\right)\sqrt{{\left(DF_{0}^{e^{\prime }}\left(1,\zeta \right)\right)}^{2}+{\left(DF_{1}^{e^{\prime }}\left(1,\zeta \right)\right)}^{2}}d\zeta +\\ +\int_{-1}^{1}g_{2}^{e^{\prime }}\cdot \upsilon_{h}^{e^{\prime }}\left(F^{e^{\prime }}\left(\zeta ,1\right)\right)\sqrt{{\left(DF_{0}^{e^{\prime }}\left(\zeta ,1\right)\right)}^{2}+{\left(DF_{1}^{e^{\prime }}\left(\zeta ,1\right)\right)}^{2}}d\zeta +\\ +\int_{-1}^{1}g_{3}^{e^{\prime }}\cdot \upsilon_{h}^{e^{\prime }}\left(F^{e^{\prime }}\left(-1,\zeta \right)\right)\sqrt{{\left(DF_{0}^{e^{\prime }}\left(-1,\zeta \right)\right)}^{2}+{\left(DF_{1}^{e^{\prime }}\left(-1,\zeta \right)\right)}^{2}}d\zeta \end{array}$$

Let *N* and *Z* represent the number of all nodes in the global finite element mesh and the number of the assumed (i.e., zeros) displacement degrees of freedom, respectively.

The substitution of Equations (67)–(69) into the variational Equation (66) results in the set of linear equations B T=Q representing the equilibrium conditions, where $B\in R^{2N\times 3N}$ is the rectangular 2*N* × 3*N* statics matrix with 2*N* rows (equal to the total number of displacement degrees of freedom) and 3*N* columns (equal to the total number of stress nodal components), $Q\in R^{2N}$ is the vector of all known nodal loads and unknown nodal reactions and $T={\left[\begin{array}{cccc} \tau_{0} & \tau_{1} & \ldots \;\tau_{j}\;\ldots & \tau_{3N-1} \end{array}\right]}^{T}\in R^{3N}$ is the vector of all unknown nodal stresses $\tau_{j}$ defining the components of the stress tensor, respectively. Similar to the methodology of the Force Method (well known in classical Structure Mechanics) used in the analysis of statically indeterminate bar structures, we perform the partition of the rectangular matrix **B** and vector **Q** into two matrices, upper $B^{u}\in R^{\left(2N-Z\right)\times 3N}$ and lower $B^{l}\in R^{Z\;\times \;3N}$, and two vectors, upper $Q^{u}\in R^{2N-Z}$ (with known components) and lower $Q^{l}\in R^{Z}$ (with unknown components), respectively. The (2*N* − *Z*) indices of the rows in upper matrix $B^{u}$ correspond to the indices of the global, unknown, free displacement degrees of freedom, and the remaining *Z* indices of the rows in lower matrix $B^{l}$ correspond to the indices of the global, known, constrained degrees of freedom (corresponding to the boundary conditions). All components of the vector $Q^{u}$ are known and the vector of the unknown boundary reactions can be calculated from the relation $Q^{l}=B^{l}\;T$ upon finding the vector T from the system of rectangular linear equations $B^{u}\;T=Q^{u}$. The set of all solutions of the equations $B^{u}\;T=Q^{u}$ can be expressed as:

$$\Theta =\left\{T=T\left(\alpha_{0},\ldots ,\alpha_{s-1}\right)|\;T=T^{*}+\sum_{k=0}^{s-1}\alpha_{k}\;T_{k},\;\alpha_{k}\in R\right\}$$

where $T_{k}={\left[\begin{array}{cccc} \tau_{0k} & \tau_{1k} & \ldots \;\tau_{jk}\;\ldots & \tau_{\left(3N-1\right)k} \end{array}\right]}^{T}\in R^{3N},\;k=0,1,\ldots ,s-1$ are the vectors that span the *s*-dimensional kernel of the matrix $B^{u}$ and $T^{*}={\left[\begin{array}{cccc} \tau_{0}^{*} & \tau_{1}^{*} & \ldots \;\tau_{j}^{*}\;\ldots & \tau_{3N-1}^{*} \end{array}\right]}^{T}\in R^{3N}$ is the arbitrary, fundamental solution of the set of linear equations $B^{u}\;T=Q^{u}$. In each *e*-th finite element $\Omega_{e}$, the stress components (see Equation (71)) depend not only on ξ∈ω (i.e., $x=F^{e}\left(\xi \right)\in \Omega_{e}$) and on appropriately selected 3*m* + 3 indices $i_{j}$, j=0,…,3m+2 (from among all 3*N* indices {0,1,…,3N−1}) defining local nodal stresses $\tau_{i_{j}}$ in *e*-th finite element, but additionally on *s* global parameters $\alpha_{k}\;,\;k=0,\ldots ,s-1$ defining the linear combinations of the *s* base vectors $T_{k}$. In other words, upon constructing the solution (found only once) of linear, rectangular algebraic system $B^{u}\;T=Q^{u}$, one obtains a very simple approximation $\Sigma_{h}^{\alpha }$ of the statically admissible set of the stress fields Σ(Ω) determined by *s* global parameters $\alpha_{k}\in R$

(70) $$\Sigma_{h}^{\alpha }=\left\{\tau_{h}=\tau_{h}\left(\alpha \right)\in S^{3}|\alpha =\left(\alpha_{0},\ldots ,\alpha_{s-1}\right)\in R^{s}\right\},$$

where in *e*-th finite element $\Omega_{e}$ the following interpolations of the stress components hold:

(71) $$\begin{array}{l} \tau_{h00}^{e}\left(\alpha \right)=\tau_{i_{0}}^{*}\;\phi_{0}\left(\xi \right)+\tau_{i_{3}}^{*}\;\phi_{1}\left(\xi \right)+\ldots +\tau_{i_{3m+0}}^{*}\;\phi_{m}\left(\xi \right)+\\ \;+\sum_{k=0}^{s-1}\alpha_{k}\;\tau_{i_{0}k}\;\phi_{0}\left(\xi \right)+\sum_{k=0}^{s-1}\alpha_{k}\;\tau_{i_{3}k}\;\phi_{1}\left(\xi \right)+\ldots +\sum_{k=0}^{s-1}\alpha_{k}\;\tau_{i_{\left(3m+0\right)}k}\;\phi_{m}\left(\xi \right)\\ \tau_{h11}^{e}\left(\alpha \right)=\tau_{i_{1}}^{*}\;\phi_{0}\left(\xi \right)+\tau_{i_{4}}^{*}\;\phi_{1}\left(\xi \right)+\ldots +\tau_{i_{3m+1}}^{*}\;\phi_{m}\left(\xi \right)+\\ \;+\sum_{k=0}^{s-1}\alpha_{k}\;\tau_{i_{1}k}\;\phi_{0}\left(\xi \right)+\sum_{k=0}^{s-1}\alpha_{k}\;\tau_{i_{4}k}\;\phi_{1}\left(\xi \right)+\ldots +\sum_{k=0}^{s-1}\alpha_{k}\;\tau_{i_{\left(3m+1\right)}k}\;\phi_{m}\left(\xi \right)\\ \tau_{h01}^{e}\left(\alpha \right)=\tau_{i_{2}}^{*}\;\phi_{0}\left(\xi \right)+\tau_{i_{5}}^{*}\;\phi_{1}\left(\xi \right)+\ldots +\tau_{i_{3m+2}}^{*}\;\phi_{m}\left(\xi \right)+\\ \;+\sum_{k=0}^{s-1}\alpha_{k}\;\tau_{i_{2}k}\;\phi_{0}\left(\xi \right)+\sum_{k=0}^{s-1}\alpha_{k}\;\tau_{i_{5}k}\;\phi_{1}\left(\xi \right)+\ldots +\sum_{k=0}^{s-1}\alpha_{k}\;\tau_{i_{\left(3m+2\right)}k}\;\phi_{m}\left(\xi \right)\\ \tau_{h10}^{e}\left(\alpha \right)=\tau_{h01}^{e}\left(\alpha \right),\;i_{j}=i_{j}\left(e\right),\;j=0,1,\ldots ,3m+2 \end{array}$$

## 6. Construction of the Approximate Solutions to the Problem (*P*) and Recovery of the Optimum Properties of the Initial Problem

The test fields τ∈Σ(Ω) of the problem (*P*) are interpolated by Equation (71) element-wise. These interpolations are ***x***-dependent, which is underlined by now using the notation $\tau_{h}\left(x,\alpha \right)$. 

Let us re-write Equation (23) in the form:

(72) $$\gamma \left(\tau \left(x\right)\right)=\sqrt{\tau_{00}^{2}\left(x\right)-\tau_{00}\left(x\right)\tau_{11}\left(x\right)+\tau_{11}^{2}\left(x\right)+3\tau_{01}^{2}\left(x\right)}\leq \sigma_{0}.$$

According to the assumed stress field interpolation (Equation (71)), the discretized version of the problem (*P*) reads: find $\alpha^{*}\in R^{s}$ such that:

(73) $$\begin{array}{l} \int_{\Omega }\;\rho \left(\tau_{h}\left(x,\alpha^{*}\right)\right)dx_{0}dx_{1}=\\ =\underset{\alpha \in R^{s}}{\min}\left\{\int_{\Omega }\;\rho \left(\tau_{h}\left(x,\alpha \right)\right)dx_{0}dx_{1}\;\mathrm{and}\;\forall x\in \Omega \;\gamma \left(\tau_{h}\left(x,\alpha^{*}\right)\right)\leq \sigma_{0}\right\} \end{array}(P_{h})$$

Integration in Equation (73) is performed numerically on master element ω, i.e.:

(74) $$\int_{\Omega }\;\rho \left(\tau_{h}\left(x,\alpha \right)\right)dx_{0}dx_{1}≅\sum_{e}\;\sum_{\begin{array}{l} \xi \in \omega \\ \xi \;\mathrm{being}\\ \mathrm{Gauss}\\ \mathrm{points} \end{array}}\;w\left(\xi \right)\;\rho \left(\tau_{h}^{e}\left(\xi ,\alpha \right)\right)\left|\det DF^{e}\left(\xi \right)\right|,$$

where here $\xi =\left(\xi_{0},\xi_{1}\right)\in \omega$ and w=w(ξ) are Gauss integration points and weights, respectively. In arbitrary element *e* and at arbitrary but fixed point ξ∈ω, the gradient:

(75) $$\nabla \rho \left(\tau_{h}^{e}\left(\xi ,\alpha \right)\right)={\left[\begin{array}{cccc} \frac{\partial \rho \left(\tau_{h}^{e}\left(\xi ,\alpha \right)\right)}{\partial \alpha_{0}} & \frac{\partial \rho \left(\tau_{h}^{e}\left(\xi ,\alpha \right)\right)}{\partial \alpha_{1}} & \ldots & \frac{\partial \rho \left(\tau_{h}^{e}\left(\xi ,\alpha \right)\right)}{\partial \alpha_{s-1}} \end{array}\right]}^{T}\in R^{s}$$

of the function $R^{s}∍\alpha \to \rho \left(\tau_{h}^{e}\left(\xi ,\alpha \right)\right)\in R$ appearing in the mapping:

(76) $$\begin{array}{l} \Pi_{h}:R^{s}\to R\\ \forall \alpha \in R^{s}\;\Pi_{h}\left(\alpha \right)=\sum_{e}\sum_{\xi \in \omega }w\left(\xi \right)\;\rho \left(\tau_{h}^{e}\left(\xi ,\alpha \right)\right)\;\left|\det DF^{e}\left(\xi \right)\right| \end{array}$$

can be computed by the rule:

(77) $$\begin{array}{l} \nabla \rho \left(\tau_{h}^{e}\left(\xi ,\alpha \right)\right)=\left[\begin{array}{c} \ldots \\ t\frac{\mathrm{tr}\;\tau_{h}^{e}\;\mathrm{tr}\;\frac{\partial \;\tau_{h}^{e}}{\partial \;\alpha_{k}}}{\sqrt{{\left(\mathrm{tr}\;\tau_{h}^{e}\right)}^{2}}}+c\frac{\left(\tau_{h}^{e}\cdot \frac{\partial \;\tau_{h}^{e}}{\partial \;\alpha_{k}}-\frac{1}{2}\mathrm{tr}\;\tau_{h}^{e}\;\mathrm{tr}\;\frac{\partial \;\tau_{h}^{e}}{\partial \;\alpha_{k}}\right)}{\sqrt{\tau_{h}^{e}\cdot \tau_{h}^{e}-\frac{1}{2}{\left(\mathrm{tr}\;\tau_{h}^{e}\right)}^{2}}}\\ \ldots \end{array}\right]\in R^{s}\\ \left(k=0,\ldots ,s-1\right),\;\left(t=1/\sqrt{2}\;,\;c=\sqrt{2}\right) \end{array}$$

where:

(78) $$\frac{\partial \;\tau_{h}^{e}}{\partial \;\alpha_{k}}=\left[\begin{array}{cc} \frac{\partial \tau_{h00}^{e}\left(\xi ,\alpha \right)}{\partial \alpha_{k}} & \frac{\partial \tau_{h}^{e}{}_{01}\left(\xi ,\alpha \right)}{\partial \alpha_{k}}\\ \frac{\partial \tau_{h10}^{e}\left(\xi ,\alpha \right)}{\partial \alpha_{k}} & \frac{\partial \tau_{h11}^{e}\left(\xi ,\alpha \right)}{\partial \alpha_{k}} \end{array}\right]$$

and:

(79) $$\begin{array}{l} \frac{\partial \tau_{h00}^{e}\left(\xi ,\alpha \right)}{\partial \alpha_{k}}=\tau_{i_{0}k}\;\phi_{0}\left(\xi \right)+\tau_{i_{3}k}\;\phi_{1}\left(\xi \right)+\ldots +\tau_{i_{\left(3m+0\right)}k}\;\phi_{m}\left(\xi \right)\\ \frac{\partial \tau_{h11}^{e}\left(\xi ,\alpha \right)}{\partial \alpha_{k}}=\tau_{i_{1}k}\;\phi_{0}\left(\xi \right)+\tau_{i_{4}k}\;\phi_{1}\left(\xi \right)+\ldots +\tau_{i_{\left(3m+1\right)}k}\;\phi_{m}\left(\xi \right)\\ \frac{\partial \tau_{h01}^{e}\left(\xi ,\alpha \right)}{\partial \alpha_{k}}=\tau_{i_{2}k}\;\phi_{0}\left(\xi \right)+\tau_{i_{5}k}\;\phi_{1}\left(\xi \right)+\ldots +\tau_{i_{\left(3m+2\right)}k}\;\phi_{m}\left(\xi \right)\\ \frac{\partial \tau_{h10}^{e}\left(\xi ,\alpha \right)}{\partial \alpha_{k}}=\frac{\partial \tau_{h01}^{e}\left(\xi ,\alpha \right)}{\partial \alpha_{k}},\;k=0,1,\ldots ,s-1,\;i_{j}=i_{j}\left(e\right) \end{array}$$

Equations (77)–(79) make it possible to calculate the quantity $\Pi_{h}$ given by Equation (76) and *s* components of its gradient for arbitrary design parameter $\alpha \in R^{s}$, i.e.:

(80) $$\begin{array}{l} R^{s}∍\alpha \to \nabla \sum_{e}\sum_{\xi \in \omega }w\left(\xi \right)\;\rho \left(\tau_{h}^{e}\left(\xi ,\alpha \right)\right)\;\left|\det DF^{e}\left(\xi \right)\right|=\\ =\sum_{e}\sum_{\xi \in \omega }w\left(\xi \right)\;\nabla \rho \left(\tau_{h}^{e}\left(\xi ,\alpha \right)\right)\;\left|\det DF^{e}\left(\xi \right)\right|\in R^{s} \end{array}$$

In arbitrary element *e*, at arbitrary point $x\in \Omega_{e}$ and for arbitrary $\alpha \in R^{s}$, let us rewrite Equation (23) as:

(81) $$\forall x\in \Omega \;\Gamma_{x}^{e}\left(\alpha \right)\leq 0,\;\;\Gamma_{x}^{e}\left(\alpha \right)=\frac{1}{\sigma_{0}}\gamma \left(\tau_{h}^{e}\left(x,\alpha \right)\right)-1.$$

In arbitrary element *e* and at point $x=F^{e}\left(\xi \right)\in \Omega_{e}$ where ξ∈ω is arbitrary, the partial derivative of Equation (81) with respect to $\alpha_{k}$ is equal to:

(82) $$\frac{\partial \Gamma_{x}^{e}\left(\alpha \right)}{\partial \alpha_{k}}=\frac{1}{\gamma \left(\tau_{h}^{e}\left(x,\alpha \right)\right)}\left(\tau_{h00}^{e}\frac{\partial \tau_{h00}^{e}}{\partial \alpha_{k}}-\frac{1}{2}\left(\frac{\partial \tau_{h00}^{e}}{\partial \alpha_{k}}\tau_{h11}^{e}+\tau_{h00}^{e}\frac{\partial \tau_{h11}^{e}}{\partial \alpha_{k}}\right)+\tau_{h11}^{e}\frac{\partial \tau_{h11}^{e}}{\partial \alpha_{k}}+3\tau_{h01}^{e}\frac{\partial \tau_{h01}^{e}}{\partial \alpha_{k}}\right),$$

where $\tau_{hij}^{e}=\tau_{hij}^{e}\left(\xi ,\alpha \right)\;\left(i,j=0,1\right)$. For arbitrary *p* > 1, let us define the function:

(83) $$\varphi :R\to R\;;\;\varphi \left(y\right)=\{\begin{array}{c} 0\;\mathrm{if}\;y\leq 0\\ y^{p}\;\mathrm{if}\;y>0 \end{array}$$

and write its derivative:

(84) $$D\varphi :R\to R\;;\;D\varphi \left(y\right)=\{\begin{array}{c} 0\;\;\mathrm{if}\;y\leq 0\\ p\;y^{p-1}\;\mathrm{if}\;y>0 \end{array}$$

In the algorithm for the numerical solution of the (P_h_) problem proposed below, we assume that the yield condition in Equation (81) is satisfied at a finite number of points, i.e., at all Gaussian points. For this reason, we slightly modify the notation of the functional in Equation (81) and replace the lower index **x** symbolizing any point in Ω with a subscript denoting the successive Gaussian points counted in subsequent finite elements $\Omega_{e}$, *e* = 0, 1, …, *M* − 1, i.e.:

(85) $$\Gamma_{g}^{e}\left(\alpha \right)=\frac{\gamma \left(\tau_{h}^{e}\left(x_{g},\alpha \right)\right)}{\sigma_{0}}-1,\;g=0,\;1,\ldots .,\;G-1,\;$$

where $x_{g}=F^{e}\left(\xi \right)\in \Omega_{e}$ is the *g*-th image of the Gauss point ξ∈ω in the master element. The index *g* runs from 0 to *G* = *m* × *M* − 1, where *m* represents the number of Gauss points in ω. We will also omit the superscript *e* identifying the number of finite elements. Now, we are ready to formulate the algorithm for solving the (*P*_h_) problem:

Step 0. Find a solution **T** of the static problem $B^{u}\;T=Q^{u}$.

From now, the design parameter is the vector $\alpha ={\left[\alpha_{0}\;\alpha_{1}\;\ldots \;\alpha_{s-1}\right]}^{T}\in R^{s}$.

Step 1. For arbitrary real number *P* > 0, define the following penalty function:

(86) $$f:R^{s}\to R\;;\;\forall \alpha \in R^{s}\;f\left(\alpha \right)=\Pi_{h}\left(\alpha \right)+P\;k\left(\alpha \right),\;$$

where $k\left(\alpha \right)=\sum_{g=0}^{G-1}\left(\varphi \circ \Gamma_{g}\right)\left(\alpha \right)$, and its gradient reads:

(87) $$\nabla f:R^{s}\to R^{s}\;;\;\forall \alpha \in R^{s}\;\nabla f\left(\alpha \right)=\nabla \Pi_{h}\left(\alpha \right)+P\;\nabla k\left(\alpha \right)\in R^{s}$$

where:

$$\nabla k\left(\alpha \right)=\;\sum_{g=0}^{G-1}D\varphi \left(\Gamma_{g}\left(\alpha \right)\right)\nabla \Gamma_{g}\left(\alpha \right)\in R^{s}.$$

Step 2. Initialize (small) real numbers: accuracy *ε* > 0, multiplier *χ* > 1, exponent *p* > 1, penalty *P* > 0.

Step 3. Initialize design parameter $\alpha_{0}$. 

Step 4. Starting with $\alpha =\alpha_{0}$, apply any algorithm of the nonlinear mathematical programming to find the solution $\alpha^{*}=\underset{\alpha \in R^{s}}{\arg\min}f\left(\alpha \right)\in R^{s}$ of the unconstrained problem $f^{*}=\underset{\alpha \in R^{s}}{\min}\;f\left(\alpha \right)$, where the function f(α) and its gradient ∇ f(α) are defined by Equations (86) and (87), respectively.

Step 5. If $P\;k\left(\alpha^{*}\right)<\varepsilon$ then STOP, otherwise calculate the new value of the penalty parameter as *P* = *χ P* and initialize design parameter $\alpha_{0}=\alpha^{*}$. Go to Step 4.

The approximants of the problem (*P*) (see Equation (33)) computed by the above algorithm will be denoted by Π^*^. The quantity Y^*^ will represent approximants of the optimal compliance Y (see Equation (32)).

## 7. Case Studies and Discussion

In the analysis of plate structures loaded in the plane, deforming within the linear elastic range, it is impossible to prevent singularities of stresses around critical points or along some lines. These points are reentrant corners, places where the load is concentrated or where the boundary conditions change abruptly and the structure loses its support. One can achieve better control over the stress level if the structure is not supported and the load is self-equilibrated; however, such problems are usually not practical. The stress-based LCP problem of the IMD method within the elastic range (the specific case of problem (*P*), Equation (33), with the yield condition being neglected) also suffers from the drawback of the possible appearance of stress singularities. Thus, according to Equation (35), the optimal moduli blow up at these places. To be more precise, the bulk modulus becomes infinite where the trace of stress tensor is singular; the shear modulus blows up where the norm of the stress deviator tends to infinity. In the plane stress problem considered, the HMH condition assumes the form of Equation (15). That is why introduction of the yield condition (Equation (23)) alleviates all components of stress. Thus, one can expect that the condition (Equation (23)) in the IMD setting should bring about cutting all extremes of all components of the stress field solving the auxiliary problem (*P*), hence making regular all layouts of the optimal elastic moduli.

**Figure 5:**
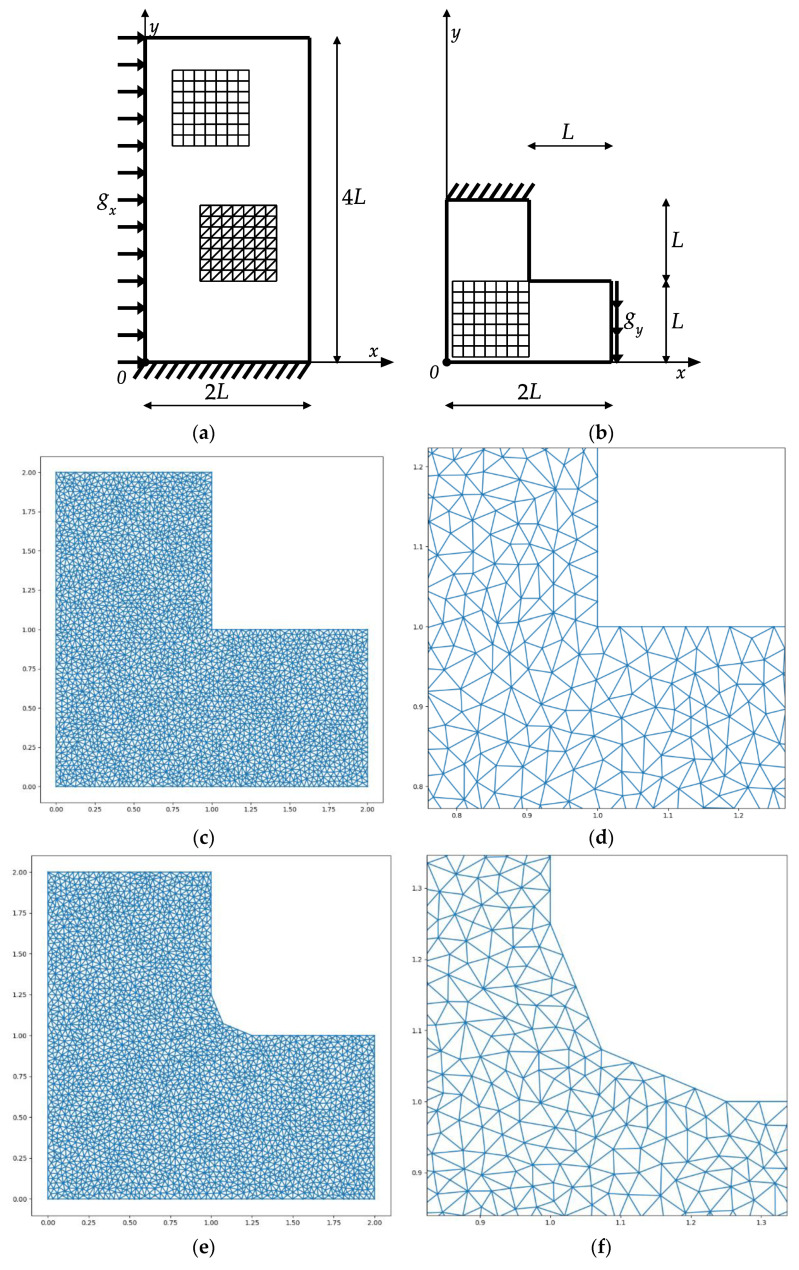
The vertical cantilever (**a**) covered by the regular quadrilateral and triangular FEM meshes and **L**-shaped cantilever (**b**) covered by the regular quadrilateral FEM mesh. The irregular FEM meshes (**c**,**e**) of triangular finite elements along with the enlarged fragments (**d**,**f**) around the reentrant corner covering two variants of the **L**-shaped cantilevers, respectively.

Two optimum design problems are considered:

- Designing the material layout within the rectangular cantilever plate (of the in-plane dimensions 2L by 4L, see Figure 5a) subjected to a lateral constant traction of intensity g_x_: Examples 1a, 1b, 1c;
- The optimum design of the L-shaped plate, see Figure 5b, subjected to the vertical shearing traction along one vertical side: Examples 2, 3.

Within the purely elastic IMD method, the optimum cantilever plate suffers singular layouts of the moduli around the left and right ends of the support. Due to the linear elastic approach, the moduli are proportional to the magnitude of the load, while the shape of the layout is load-independent. The plastic version of the IMD introduces an essential change: the optimal layout of the moduli does depend upon the ratio: $g_{x}/\sigma_{0}$, hence the layout of the optimal moduli becomes dependent on the magnitude of the load. We also have control over the size of plastic zones. One of the aims of the present paper is to analyze sequences of the optimal designs corresponding to various values of the ratio $g_{x}/\sigma_{0}$.

The optimal designs of the rectangular cantilever plate have been constructed by using the special software based on the numerical scheme outlined in Section 5. Two kinds of finite elements are used: the triangular (T) and quadrilateral (Q) described in Section 5. Both FE meshes are regular.

The same software has been used to design the optimal moduli within: the L-shaped plate of sharp corners (see Figure 5b,c) and within the L-shaped plate with the reentrant corner being slightly rounded (see Figure 5e). The plate in Figure 5b is meshed by quadrilateral finite elements; the mesh is regular. The meshes for the plate in Figure 5c,e are irregular and composed of triangular finite elements.

Let us fix the data: the thickness *b* of the plate and the length L=1.0 m. The material to be designed is viewed as a composite of the properties varying around the values of the physical characteristics of aluminum. Thus, in all examples the referential Young modulus and the Poisson ratio are assumed to be equal: E=72000.0 MPa, ν=0.34, respectively. The yield stress will be fixed as: $\sigma_{0}^{3D}=\sigma_{0}/b=50.0\;MPa$. Let us note that the yield stress $\sigma_{0}$ has the units N/m, like the units of the stress resultants in the in-plane loaded plate. The value of the referential modulus *E*_0_ (this is not Young’s modulus, its units are N/m) appearing in the isoperimetric condition (Equation (27)) is assumed now as *E*_0_ = 2*k*_0_ + 4*µ*_0_, where:

(88) $$k_{0}=\frac{Eb}{2\left(1-\nu \right)}\;,\;\mu_{0}=\frac{Eb}{2\left(1+\nu \right)}$$

are characteristic bulk and shear stiffnesses of the plate of thickness *b,* made of the referential homogeneous material with moduli E, ν. The values of the remaining parameters appearing in the penalty function algorithm are adopted as follows:

$$\begin{array}{l} accuracy\;\varepsilon =5.0\times 10^{-4},\;multiplier\;\chi =1.3\\ exponent\;p=2,\;initial\;penalty\;P=1.0\times 10^{-2},\;ftol=1.0\times 10^{-5} \end{array}$$

The last quantity *ftol* is a parameter used in the gradient-oriented *frprmn*(…) procedure in C++ (see [27] implementing the Fletcher–Reeves–Polak–Ribiere algorithm of the minimization of functions without constraints). Numerical integration has been performed for the master element on the basis of the rules of integration with one and four Gauss points for triangular (T) and quadrilateral (Q) finite elements, respectively. All the data are now given, and the results are ready to be replicated.

**Example** **1.** The optimum design of the rectangular cantilever plate.

**Case** **1a.** The lateral horizontal traction of intensity *g_x_* = 0.01 ∙ *σ*_0_ applied to the left edge (see Figure 5a).

**Figure 6:**
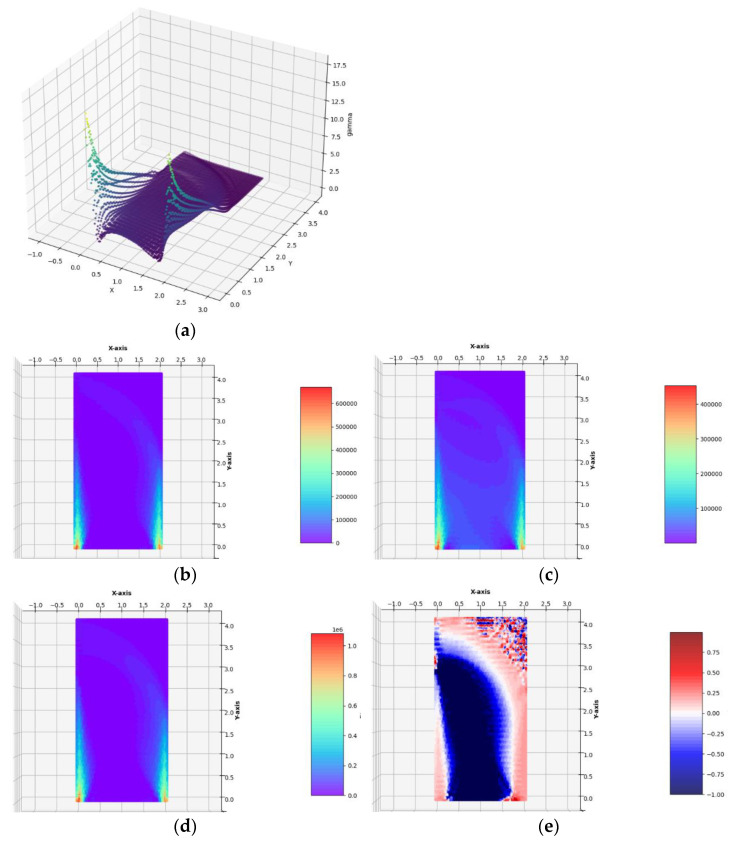
Optimal solutions of the problem of Example 1a. (**a**) The plot of the *γ* function, (**b**) bulk modulus k^*^/*b*, (**c**) shear modulus µ^*^/*b*, (**d**) Young’s modulus E^*^/*b*, (**e**) Poisson’s ratio ν^*^ in the case of FEM mesh composed of quadrilateral elements.

**Figure 7:**
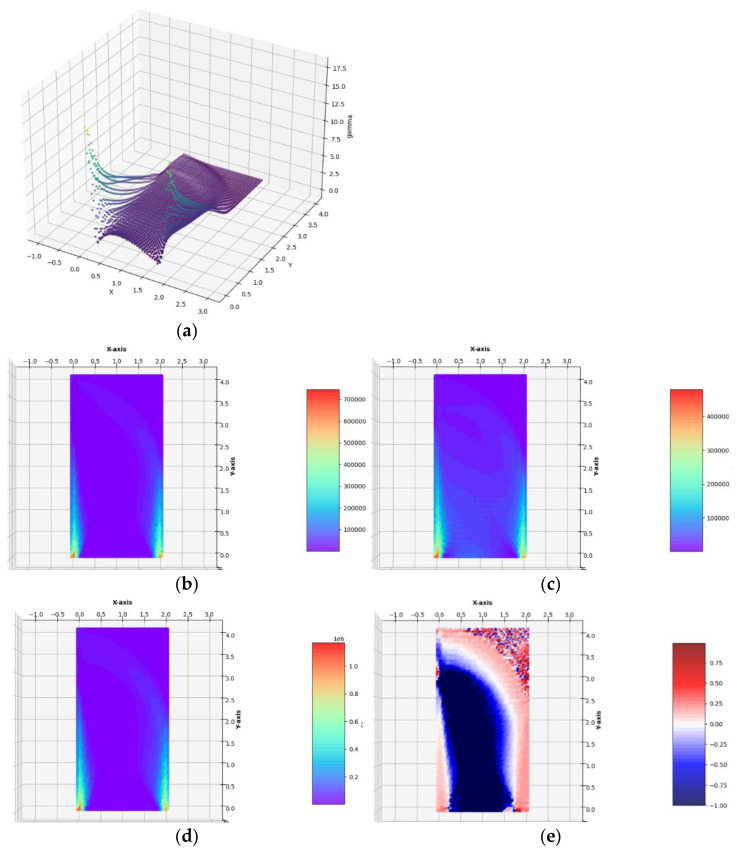
Optimal solutions of the problem of Example 1a. (**a**) The plot of the *γ* function, (**b**) bulk modulus k^*^/*b*, (**c**) shear modulus µ^*^/*b*, (**d**) Young’s modulus E^*^/*b*, (**e**) Poisson’s ratio ν^*^ in the case of FEM mesh composed of triangular elements.

The optimum design problem (Equation (28)) has been solved by applying the numerical method outlined in Section 5 and Section 6. The two regular FEM meshes composed of 34 × 69 = 2346 quadrilateral and 68 × 69 = 4692 triangular finite elements were used. It has occurred that for sufficiently dense FEM meshes, the results obtained for triangular and quadrilateral elements are practically identical (see Figure 6 and Figure 7). For this reason, the next results of optimal distributions of elastic moduli will be presented for a mesh spanned only by quadrilateral or only by triangular finite elements.

The optimal layouts of the moduli $k^{*},\;\mu^{*}$ have been constructed by Equation (35), and the moduli $E^{*},\;\nu^{*}$ are computed by:

(89) $$E^{*}=\frac{4k^{*}\mu^{*}}{k^{*}+\mu^{*}},\;\nu^{*}=\frac{k^{*}-\mu^{*}}{k^{*}+\mu^{*}}$$

(see Figure 6 and Figure 7). Because the traction is small, Equation (23) does not introduce essential cutting of the plot of γ(σ). The final numerical results are:

Optimal compliance Y^*/^*b* = 0.0001769 MN and П^*^/*b* = 17.509 MN: mesh (Q)

Optimal compliance Y^*^/*b* = 0.0001764 MN and П^*^/*b* = 17.483 MN: mesh (T)

**Case** **1b.** The lateral traction of intensity *g_x_* = 0.1 ∙ *σ*_0_ applied to the left edge.

**Figure 8:**
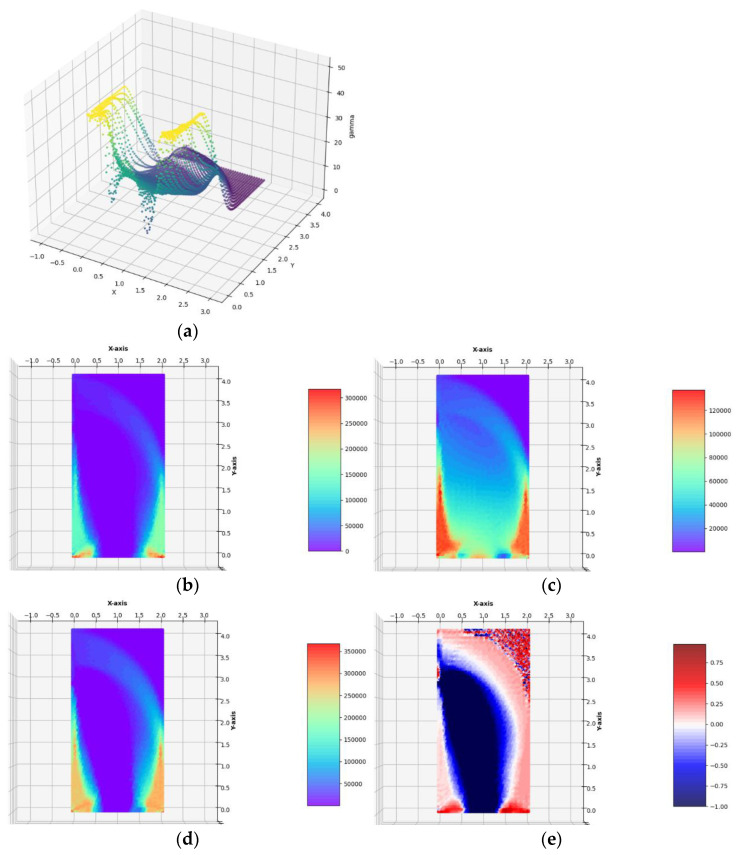
Optimal solutions for the Example 1b. (**a**) The plot of the *γ* function, (**b**) bulk modulus k^*^/*b*, (**c**) shear modulus µ^*^/*b*, (**d**) Young’s modulus E^*^/*b*, (**e**) Poisson’s ratio ν^*^ in the case of FEM mesh composed of triangular elements.

The optimal layouts of the moduli $k^{*},\;\mu^{*}$, $E^{*},\;\nu^{*}$ have been constructed (see Figure 8). The final numerical results are:

Optimal compliance Y^*^/*b* = 0.0197369 MN and П^*^/*b* = 184.913 MN: mesh (Q)

Optimal compliance Y^*^/*b* = 0.0196817 MN and П^*^/*b* = 184.654 MN: mesh (T)

**Figure 9:**
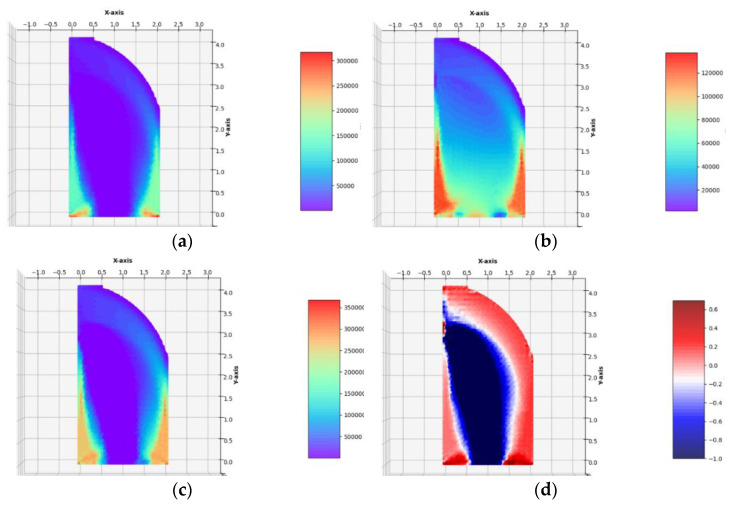
Optimal solutions for the Example 1b. In (**a**–**d**), the optimal layouts of the bulk, shear and Young moduli k^*^/*b*, µ^*^/*b*, E^*^/*b* and of Poisson’s ratio ν^*^, with the upper right corner of Ω being cut off, in the case of FEM mesh composed of triangular elements.

Zero (or numerically close to zero) value*s* of the optimal moduli k^*^ and µ^*^ mean in practice the need to cut off these sub-areas from the entire Ω domain. In Figure 9 the same as in Figure 8, the optimal distributions of elastic moduli are shown with a clearly visible modification of the optimal shape of Ω consisting of cutting off the right upper corner of the cantilever at all those points where both optimal values of k^*^ and µ^*^ are equal to zero or are numerically close to zero. However, the correct cutting off of the material inside the design domain cannot be easy programmed. For this reason, in the further examples, the empty domain within the design domain will not be cut off.

**Case** **1c.** The horizontal lateral traction of intensity *g_x_* = 0.125 ∙ *σ*_0_ applied to the left vertical edge.

**Figure 10:**
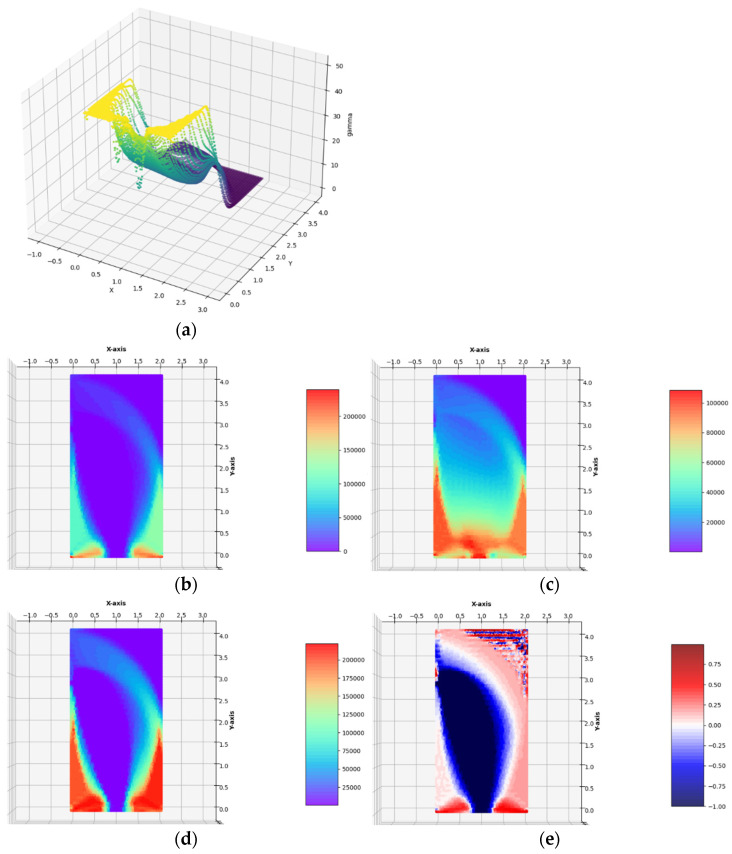
Optimal solutions of the problem of Example 1c. (**a**) The plot of the *γ* function, (**b**) bulk modulus k^*^/*b*, (**c**) shear modulus µ^*^/*b*, (**d**) Young’s modulus E^*^/*b*, (**e**) Poisson’s ratio ν^*^ in the case of FEM mesh composed of quadrilateral elements.

The optimal layouts of the moduli $k^{*},\;\mu^{*}$, $E^{*},\;\nu^{*}$ have been constructed (see Figure 10). The final numerical results are:

Optimal compliance Y^*/^*b =* 0.0335001 MN and П^*^/*b* = 240.908 MN: mesh (Q)

Optimal compliance Y^*^/*b* = 0.0334105 MN and П^*^/*b* = 240.585 MN: mesh (T)

Having constructed the optimal designs for three subsequent, increasing magnitudes of the lateral traction, one can discuss the influence of the parameter *g*_x_/*σ*_0_ on the final solutions. Along with the increase in the lateral load *g*_x_, one can observe that those zones of the design domain Ω expand, in which the optimal moduli k^*^ and µ^*^ assume high or moderate values; those zones are shown in orange and red. This is very visible while comparing the layouts of the moduli k^*^ and µ^*^ in the vicinity of the lower vertices of the design domain, along the lower horizontal edge and both the vertical edges. In the case of the small load, which does not induce the plastic zones within the design domain, making the mesh denser causes the shrinking of the zones of high values of the optimal elastic moduli (see Figure 6) to several finite elements (whose dimensions are smaller and smaller if the mesh is made denser) around the corners. Just in these elements, the values of the optimal moduli grow up, thus making the cost condition satisfied. These values tend to infinity along with making the mesh denser and denser. By the introduction of the plastic limit within the whole design domain, we ban the mentioned tendency to accumulate the high values of the optimal moduli around some points; the zones of high values of the moduli become broader along with the expansion of plastic zones. This tendency is easy to verify by comparing the optimal layouts of the elastic moduli shown in Figure 6, Figure 7, Figure 8, Figure 9 and Figure 10. The plastic zones are places where the *γ* function attains the upper bound—see places in yellow in Figure 8, Figure 9, where the plot of the function *γ* becomes flat. Let us note that the intensity of the load can be increased only up to a certain limit; if this limit is exceeded, the problem (*P*_h_) ceases to be solvable. Moreover, it is worth stressing that in each case (presented in Figure 6, Figure 7, Figure 8, Figure 9 and Figure 10) the optimal Poisson ratio assumes the values from the whole admissible range: (−1,1); in particular, the auxetic zones (with negative Poisson ratio) appear in all cases, where necessary. In the case of the appearance of optimal plastic zones, the shape of the sub-domains in which the Poisson ratio remains negative changes slightly, always keeping the full range of its extremely small negative values. This can be partially explained by recalling the well-known properties of auxetic materials, in particular those related to the influence of negative Poisson’s ratio on the values of the stress concentration factor in the design of body components subjected to stress: “*When the Poisson’s ratio becomes negative, stress concentration factors are reduced in some situations and unchanged or increased in others*.”—see [28]. The results of many studies suggest that very often (but not always) a negative Poisson’s ratio gives the lowest possible (i.e., the most desirable) value of the stress concentration factor, which can be, in an analogous way, justified by our numerical results of optimal distributions of elastic moduli minimizing the compliance of the elasto-plastic body with a simultaneous demand to meet the Mises plasticity condition at all points within the design domain Ω. However, the study does not analyze the impact of the optimal auxetic sub-domains on the values of the stress concentration factors. Many very interesting results on this subject can be found, e.g., in the monograph [8].

**Example** **2.** Optimum design of the **L** -shaped cantilever plate (see Figure 5b–d).

**Figure 11:**
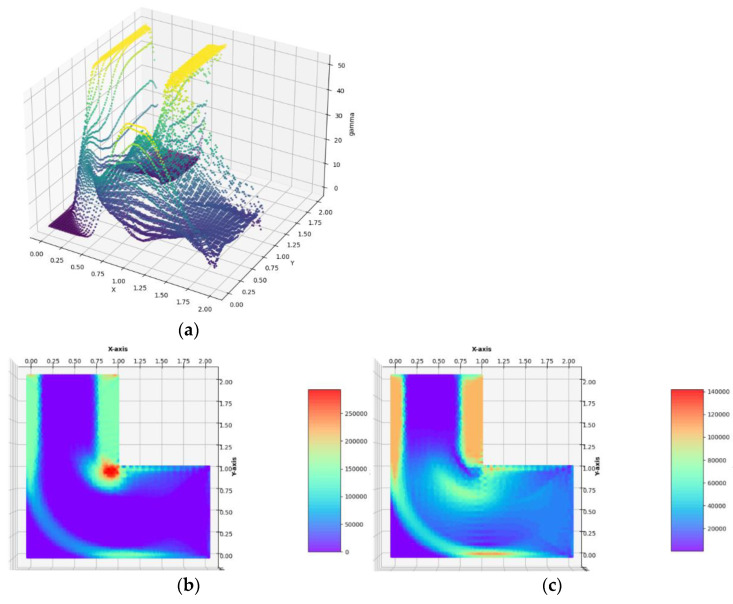

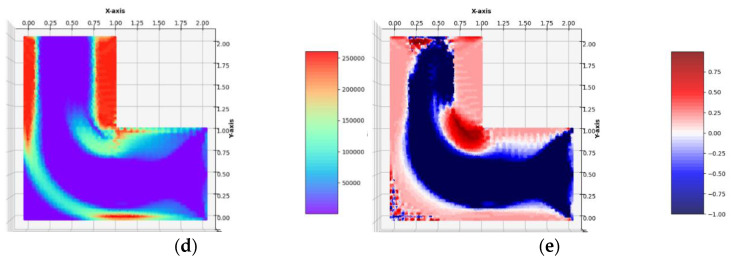
Optimal solutions of the problem of Example 2. (**a**) The plot of the *γ* function, (**b**) bulk modulus k^*^/*b*, (**c**) shear modulus µ^*^/*b*, (**d**) Young’s modulus E^*^/*b*, (**e**) Poisson’s ratio ν^*^ in the case of FEM mesh composed of quadrilateral elements.

The design problem has been solved with the use of regular and irregular FE meshes composed of 2523 quadrilateral or 5833 triangular finite elements, respectively. The **L**–shaped cantilever is loaded with the vertical tangent traction of intensity *g_y_* = 0.1 ∙ *σ*_0_ applied to the right lower vertical edge. The optimal layouts of the moduli $k^{*},\;\mu^{*}$,$E^{*},\;\nu^{*}$ have been constructed (see Figure 11). The final numerical results are:

Optimal compliance Y^*^/*b* = 0.0093363 MN and П^*^/*b* = 77.881 MN: mesh (Q)

Optimal compliance Y^*^/*b* = 0.0092781 MN and П^*^/*b* = 77.638 MN: mesh (T)

**Example** **3.** Optimum design of the **l**-shaped cantilever with a slightly rounded reentrant corner, see Figure 5e,f.

**Figure 12:**
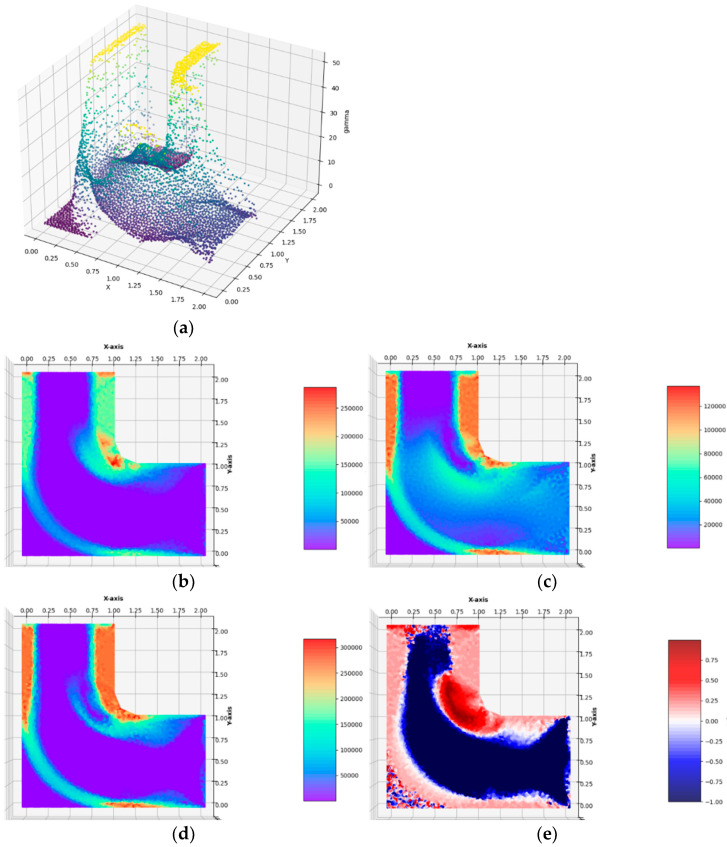
Optimal solutions of the problem of Example 3. (**a**) The plot of the *γ* function, (**b**) bulk modulus k^*^/*b*, (**c**) shear modulus µ^*^/*b*, (**d**) Young’s modulus E^*^/*b*, (**e**) Poisson’s ratio ν^*^ in the case of FEM mesh composed of triangular elements.

The plate is covered with an irregular mesh of 5803 triangular finite elements. The cantilever is loaded with the vertical tangent traction of intensity *g_y_* = 0.1 ∙ *σ*_0_ applied to the right lower vertical edge. The optimal layouts of the moduli $k^{*},\;\mu^{*}$, $E^{*},\;\nu^{*}$ have been constructed (see Figure 12). The final numerical results are:

Optimal compliance Y^*^/*b* = 0.0078393 MN and П^*^/*b* = 71.581 MN: mesh (T)

All the remarks concerning the interpretation of the results concerning the rectangular cantilever apply here. Moreover, by making the reentrant corner curve smoothly, we alleviate the stress concentration, thus making the optimal Young modulus and Poisson’s ratio layouts much more regular (see Figure 11 and Figure 12).

## 8. Conclusions

**Figure 13:**
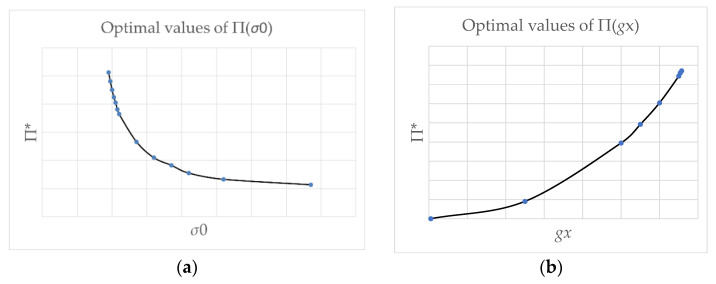
Optimal П^*^ = П^*^(σ_0_, g*_x_*) calculated for the rectangular cantilever (see Figure 5a) as a function of: (**a**) yield stress σ_0_, i.e., П^*^ = П^*^(σ_0_) (lateral load g_x_ is fixed), (**b**) intensity g_x_ of the traction load, i.e., П^*^ = П^*^(g*_x_*) (yield stress σ_0_ is fixed).

The hitherto existing works on topology optimization enhanced with local stress constraints have been formulated within the elastic range: on the stress components, being associated with the displacement field, the local constraints are imposed; they can concern all the components of stresses (see [29]) or the effective stress (see, e.g., [30]). In the present paper, another formulation of the topology optimization problem is set forth: the Hencky-Nadai-Ilyushin elasto-plastic theory is adopted in which the stress state is not linked directly with the displacement field. Thus, the optimal structure (here: an in-plane loaded plate) works within the elasto-plastic range. Consequently, the optimal design does depend upon the ratio: intensity of the load/yield stress. One of the aims of this paper is to analyze the variation of the design for a given load, if the yield stress level varies. It turns out that the approximants П^*^ of the optimal compliance calculated for subsequent values of the plasticity limit and fixed intensity of the traction load decrease with the increasing value of yield stresses σ_0_ (see Figure 13a). If for an assumed intensity of the traction load the yield stress is taken too small, it is not possible to attain the minimum П^*^ of the mapping П_h_, which means that an optimal solution does not exist. Similar conclusions hold in the case of increasing the load g_x_ for the assumed constant value of the yield stress σ_0_ (see Figure 13b).

The research planned will concern the design of the underlying microstructures exhibiting the given effective yield limit, characterized by the effective moduli predicted by the IMD method.

## Data Availability

Data sharing is not applicable to this article.
